# The Production of Intensified Qu and Its Microbial Communities and Aroma Variation during the Fermentation of Huangjiu (Chinese Rice Wine)

**DOI:** 10.3390/foods12142674

**Published:** 2023-07-11

**Authors:** Siman Zheng, Wendi Zhang, Qing Ren, Jihong Wu, Jinglin Zhang, Bowen Wang, Nan Meng, Jinchen Li, Mingquan Huang

**Affiliations:** Key Laboratory of Brewing Molecular Engineering of China Light Industry, Beijing Technology & Business University (BTBU), Beijing 100048, China; siman9912@163.com (S.Z.); 15811204457@163.com (W.Z.);

**Keywords:** Huangjiu, intensified Qu, microbial community, volatile aroma compounds, correlation analysis

## Abstract

In recent years, intensified Qu (IQ) has been gradually applied to brewing in order to improve the aroma of Huangjiu (Chinese rice wine). In this study, *Saccharomyces cerevisiae* and *Wickerhamomyces anomalus* solutions were added to Fengmi Qu (FMQ) from Fangxian, China to produce IQ, and brewing trial was conducted. High-throughput sequencing (HTS) was used to analyze the microbial community in fermentation broth of IQ (IQFB). Headspace solid-phase microextraction (HS-SPME) combined with gas chromatography–mass spectrometry (GC-MS) and sensory evaluation were performed to analyze volatile aroma compounds (VACs) in sample without Qu and both fermentation broths. The results showed that *Pediococcus*, *Cronobacter*, *Enterococcus*, *Weissella*, and *Acinetobacter* and *Saccharomycopsis*, *Wickerhamomyces*, and *Saccharomyces* were dominant bacterial and fungal groups, respectively. A total of 115 VACs were detected, and the content of esters including ethyl acetate, isoamyl acetate, and so on was noticeably higher in IQFB. The finding of sensory evaluation reflected that adding pure yeast to Qu could enhance fruit and floral aromas. Correlation analysis yielded 858 correlations between significant microorganisms and different VACs. In addition, prediction of microbial community functions in IQFB revealed global and overview maps and carbohydrate metabolism to be the main one. This study is advantageous for further regulation of the fermentation process of Huangjiu by microbial means.

## 1. Introduction

Huangjiu (Chinese rice wine), a national specialty with a long history in China, has abundant nutritional value and distinctive flavor, making it highly favored by consumers [1,2]. There are differences in residual sugar, so Huangjiu is divided into four categories including dry (≤15 g/L), semi-dry (15.1 g/L~40.0 g/L), semi-sweet (40.1 g/L~100.0 g/L), and sweet (>100 g/L) [3]. According to regional characteristics, Huangjiu is always classified into *zhepai*, *supai*, *haipai*, *peipai*, and *minpai* [4]. It is typically made of glutinous rice, millet, corn, yellow rice, and other grains as the main raw materials, saccharified by wheat Qu or xiao Qu, and fermented by yeast (*Saccharomyces cerevisiae*) [3,5]. The production of Huangjiu mainly consists of the following steps: soaking raw materials, cooking, airing, blending with Qu, saccharifying fermentation, post-fermentation, filtering, sterilization, and aging [1,6,7].

Qu is the feature of Chinese brewing and plays an important role in the saccharification and fermentation process of wine [8]. As a saccharifying and fermenting agent of Huangjiu, xiao Qu is produced by grains and subsidiary materials naturally inoculated with various microorganisms such as molds, yeasts, bacteria, and so on [9]. Research has shown that, comparing wheat Qu with commercial mixed enzyme to ferment Huangjiu, the content of esters and amino acids in the former is significantly increased and wheat Qu makes a prominent contribution to the unique aroma and flavor of Huangjiu [10]. It is the synergistic fermentation effect of microorganisms in wheat Qu during the brewing process of Huangjiu that endows it with a typical flavor. Therefore, the quality of Huangjiu could be regulated through Qu. In order to solve the problems including but not limited to an insufficient aroma attribute in Huangjiu, poor flavor, low saccharification ability, and liquor-producing power of Qu, intensified Qu (IQ) has been gradually applied to the brewing of Huangjiu. By means of microbial separation technology, strains which can enhance the performance of koji are screened out, then they are pure cultured and inoculated on koji to obtain IQ [11]. The strains used for reinforcement of Qu properities can be single or a combination of multiple strains. Wei et al. [12] screened pure *Rhizopus* from glutinous rice wine starter and added it to *Saccharomyces cerevisiae* in different proportions to manufacture fortified Qu. Compared with common commercial koji, the saccharification power and liquefaction power of fortified Qu increase 14.8% and 20.7%, respectively. After brewing with this Qu, the total ester and alcohol contents also improve 168.9% and 92.1%, respectively. Cui et al. [13] made IQ using three strains of yeasts and two strains of bacteria and added it to Zaopei to conduct enhanced fermentation experiments. The concentrations of volatile components in fermented grains including ethyl acetate and 2,3,5-trimethylpyrazine rose compared with the control group, providing a theoretical foundation for improving the quality of sauce-flavor Baijiu.

Recently, as the important quality standards for evaluating Huangjiu, aroma and flavor were researched and analyzed extensively by a great deal of scholars. Chen et al. [14] studied the differences in aroma characteristics between traditional and modern types of Huangjiu, finding that Qu, caramel, and smoky aromas of traditional Huangjiu were stronger than modern types’. In addition, the distinct contents of benzaldehyde, vanillin, sotolon, geosmin, and phenol are the significant reason for the difference in aroma between the two kinds of Huangjiu. Ye et al. [15] found that with the fermentation process, the quantities and concentrations of esters kept increasing by identifying the volatile compounds of millet Huangjiu during brewing. Moreover, 10 compounds such as ethyl acetate had the greatest aroma contribution for the final fermented millet Huangjiu and 14 compounds such as formic acid were the main substances causing diversities in aroma and flavor of millet Huangjiu at various fermentation stages. The production of volatile compounds, polyphenols, polysaccharides, proteins, and other substances in wine is closely linked with the metabolism of microorganisms [16]. As a consequence, microorganisms play a vital role in the aroma and flavor formation of Huangjiu. Yan et al. [17] explored the microorganisms producing higher alcohol in northern Huangjiu and found that *Lactobacillus*, *Neisseria*, *Staphylococcus*, *Thauera*, and *Bifidobacterium* were the functionally predominant bacteria to produce higher alcohol, which were bound up with the production of 2-methyl-1-propanol, phenethyl alcohol, 2-phenoxy-ethanol, 1-triacontanol, and 1-hexacosanol, as well as provide new ideas for controlling the generation of higher alcohol. Ren et al. [18] found that there were 557 kinds of correlations between major flavor compounds and bacteria in the Huangjiu fermented from corn. Additionally, dominant bacteria producing flavor included *Lactococcus*, *Virgibacillus*, *Sphingobacterium*, and *Sporolactobacillus*, providing reference for developing new varieties of Huangjiu.

Due to the advantages of geographical location and environmental conditions, there is a superb place called Fangxian in Shiyan City, Hubei Province, China for making Huangjiu. Therefore, fuzhi Huangjiu has become one of the local specialties. The Qu of fuzhi Huangjiu is added to a Chinese herbal medicine called polygonum, also known as JIUYAO. The JIUYAO is made of japonica rice, polygonum, high-quality JIUYAO made in previous years, and water. However, the production of JIUYAO is carried out in an open environment, where different kinds of microorganisms are enriched in the JIUYAO, resulting in its unstable quality and a plethora of miscellaneous bacteria. Consequently, on the basis of previous JIUYAO, improvements were made to develop a formula of IQ, and two types of Qu were also used for Huangjiu fermentation, respectively. Then, the fermentation broths were collected during the fermentation process and their physicochemical indexes were measured according to national standards and other methods. Next, amplicon sequencing was applied to comprehensively analyze the microbial community of the fermentation broths and headspace solid-phase microextraction (HS-SPME) combined with gas chromatography–mass spectrometry (GC-MS) was used to identify their volatile aroma compounds (VACs). The fermentation broths were also subjected to sensory evaluation. Finally, by means of a correction analysis, the relationship between dominant microorganisms and VACs in fermentation broths was established, and the metabolic pathways of microorganisms were predicted. In this study, IQ was produced by adding two types of yeast to the previously made JIUYAO to strengthen the aroma of Huangjiu, which provides a reference for stabilizing the quality of Qu and enhancing the quality of Huangjiu.

## 2. Materials and Methods

### 2.1. Preparation of Materials and Reagents

Japonica rice was obtained from COFCO Corporation. Wuchang glutinous rice was obtained from Wuchang Yinhe Rice Industry Co., Ltd., Wuchang, China. Fengmi Qu (FMQ), polygonum, bran, and rice husk were purchased from Fangxian, Shiyan City, Hubei Province, China. Glucoamylase for brewing and CICC41435 *Rhizopus oryzae* were obtained from China Industrial Microbial Strain Preservation and Management Center. Copper sulfate pentahydrate, potassium sodium tartrate, sodium hydroxide, glucose, methylene blue, hydrochloric acid, and methyl red (analytical reagents, ARs) were purchased from Sinopharm Chemical Reagent Co., Ltd., Beijing, China. 2-octanol was purchased from Beijing Bailingwei Technology Co., LTD, Beijing, China. Edible lactic acid was purchased from Henan Jindan Lactic Acid Technology Co., Ltd., Zhoukou, China.

### 2.2. Production of Intensified Qu

200 g of glutinous rice were washed and soaked for 15 h. After steaming rice, 400 mL of water and 3.0 g of glucoamylase were added, then saccharified for 3–4 h at 60 °C. After filtration, the sugar content was diluted to 14 °Bx, and then sterilized at 115 °C for 15 min to prepare rice starter juice.

30 g of rice husks were washed, moistened with boiling water, filtered dry, and mixed with 120 g of bran. 105 mL of hot water above 90 °C was added to moisten the grains for 1 h, and 7.5 mL of 14 °Bx rice starter juice was added. After mixing, they were placed in 10 triangular flasks and sterilized at 121 °C for 30 min to obtain mold expanded culture medium.

*Rhizopus oryzae* was inoculated into the expanded culture medium, cultured for 3–5 days, and mold spores were washed with sterile physiological saline. After filtering, the spore count was adjusted to 107 CFU/mL to prepare mold spore suspension.

The production method of IQ was as follows: firstly, 252 g of rice flour, 30 g of bran, 18 g of rice husk, and 1.5 g of polygonum were taken, then crushed and sieved through 60 mesh screens. Secondly, 2% FMQ, 130 mL of edible lactic acid (pH 5–5.5), and 2% *Rhizopus oryzae* spore solution were added. They were mixed well and left to stand for 1 h. Thirdly, 2% *Saccharomyces cerevisiae* solution and 1% *Wickerhamomyces anomalus* solution were added, and the water content was replenished to 60%. The above two strains were isolated and identified from JIUYAO in Fangxian by our laboratory. Fourthly, they were made into balls or blocks, coated with powder (1% FMQ powder), and then put into a plate with holes at the bottom, where two layers of dry gauze were placed. After adding the Qu, they were covered with a damp gauze cloth on the plate. Finally, in the fermentation chamber, they fermented for 24–36 h, and the temperature was controlled at 30–35 °C until the hyphae of *Rhizopus oryzae* covered the surface before removing the gauze. After being transferred to a cool and ventilated place, they were further cultured for 2–3 days to measure their moisture and saccharification power.

The saccharification power and moisture content of Qu were determined using the QB/T 4257-2011 General Analysis Method for Brewing Daqu.

### 2.3. Fermentation Process of Huangjiu

After washing 1.0 kg of glutinous rice, it was soaked for 15 h. The steamed rice was cooled to 30–35 °C in an ultraclean bench (Suzhou Antai Air Technology Co., LTD, Suzhou City, Jiangsu Province, China). Approximately 600 mL of water was poured into the glutinous rice to disperse the rice, while adding 0.7% FMQ and IQ separately. They were stirred evenly and a nest was made for the glutinous rice, which was fermented for 5 days at 30 °C. After 600 mL of sterile water was added, the fermentation broths were stirred evenly and fermented at 15 °C for 27 days. At the same time on the 3rd, 5th, 9th, 14th, 20th, and 27th days of fermentation, samples of Huangjiu fermentation mash were aspirated out. Sterile straws were used to place fermented mash into sterile centrifuge tubes. The sample before mixing Qu was recorded as Blank. Fermentation broth brewed with FMQ was labeled as FMQFB, while that brewed with IQ was labeled as IQFB. Samples were stored in a −20 °C refrigerator for analysis.

### 2.4. Determination of Physical and Chemical Indexes of Fermentation Broths

The pH value, total sugar, and total acid of Huangjiu fermentation broths were measured according to the determination methods of pH, total sugar, and total acid in the national standard GB/T13662-2018 Huangjiu.

The alcohol contents of Huangjiu fermentation broths were determined by a 1260 high-performance liquid chromatograph (Agilent Technologies, Foster City, CA, USA). To begin with, the fermentation broths were filtered through 0.45 µm polyethersulfone membranes and placed in liquid phase vials for later use. Next, 1, 2, 4, 8, 12, 16, and 32% ethanol aqueous solutions were prepared through membrane filtration to be used later.

The conditions of HPLC were set as follows: the mobile phase was ultrapure water with a flow rate of 0.8 mL/min. The detector was RID with a temperature of 55 °C. Hi Plex H (300 × 7.7 mm) was used as a chromatographic column. The temperature of the column temperature box was 70 °C.

### 2.5. DNA Extraction, PCR Amplification, and Illumina MiSeq Sequencing

The approaches for DNA extraction and amplification were consistent with a literature report [19]. A GeneAmp 9700 (ABI, Carlsbad, CA, USA) instrument was applied to perform polymerase chain reaction (PCR). Using primers 338 F and 806 R, the V3–V4 highly variable region of the 16S rRNA gene of bacteria in the fermentation broths was amplified. The amplification conditions were incubation at 95 °C for 3 min, denaturation at 95 °C for 30 s, annealing at 55 °C for 30 s, and extension at 72 °C for 45 s. The number of cycles was 27, and after the last cycle, it was kept at 72 °C for 10 min. The PCR amplification primers for the ITS region of fungi were ITS1F and ITS2. The amplification conditions are incubation at 95 °C for 3 min, denaturation at 95 °C for 30 s, annealing at 55 °C for 30 s, extension at 72 °C for 45 s. The number of cycles was 35, and finally the extension was at 72 °C for 10 min.

The purified and identified PCR products were sequenced by the Majobio Institute (Shanghai, China). The MiSeq platform of an Illumina high-throughput sequencer (Illumina, San Diego, CA, USA) was used to sequence.

### 2.6. Determination of VACs in Fermentation Broths

The VACs of fermentation broths were detected by HS-SPME combined with GC-MS. 5 mL of fermentation broth was added to a 10 mL headspace extraction bottle, mixing 2.0 g of NaCl and 0.020 mL of 2-octanol (99.07 mg/L). They were equilibrated in a water bath at 50 °C for 20 min, and then the SPME extraction head (carbon molecular sieve/polydimethylsiloxane extraction head, Sigma Aldrich, St. Louis, MO, USA; SPME manual injection handle, Ampu, Shanghai, China) was inserted into a headspace bottle. It was pushed out, extracted at 50 °C for 50 min, and then desorbed at 250 °C at the GC injection port for 5 min [20].

The instrument method of GC-MS (TRACE1310-ISQ LT, Thermo Fisher Scientific, Cambridge, MA, USA) was as follows [21]: the injector temperature was 250 °C, and the carrier gas was helium (99.999%) with a flow rate of 1.2 mL/min. There was no split flow. The compounds were separated on a DB-FFAP column (30 m × 0.25 mm × 0.25 μm, Agilent, Palo Alto, CA, USA). The initial temperature was 50 °C, maintained for 3 min, then increased from 50 °C to 130 °C at a rate of 3 °C/min, and finally increased from 130 °C to 240 °C at a rate of 6 °C/min and maintained for 10 min. The MS was run in EI + mode (70 eV) and detection was operated in scan mode over a range of 35–400 m/z. The temperatures of transmission line and ion source were 240 °C and 250 °C, respectively.

Qualitative analysis of VACs was conducted through the NIST 2017 spectral library (compounds with a retention matching degree greater than 700) and retention index (RI) [15]. The difference between the RI of VACs calculated for n-alkene (C5–C25, o2si, Charleston, SC, USA) and the RI of the NIST library was less than 50 [21]. An internal standard method was used for the quantification of the VACs [22]. All results were the average of three replicates, expressed as mean ± standard deviation.

### 2.7. Sensory Evaluation of Fermentation Broths

The sensory evaluation method for the aroma of Huangjiu fermentation broths was carried out referring to a previous study [23]. Fifteen panelists (seven males and eight females, aged between 23 and 28 years old) with experience and training in sensory evaluation were recruited from the laboratory to first score the blank sample. Then, two types of Huangjiu sample were placed in randomly coded wine glasses, and sensory evaluators were asked to rate the aroma of them. The sensory evaluation approval was granted by the Ethics Committee of Beijing Technology and Business University, and all group members signed an informed consent form before the experiment.

### 2.8. Statistical Analysis

Usearch (version 7.0.1090, www.drive5.com/usearch/, accessed on 18 May 2023) software was used to extract the high-quality sequencing readings. The RDP classifier Bayesian algorithm was conducted for taxonomy analysis of 97% similar OTU representative sequences on the Qiime platform (version 1.9.1), and the confidence threshold was 0.7.

In order to calculate the Spearman correlation coefficient between microbial genera and VACs, RStudio software (version 4.2.1) was used. A correlation network between volatile metabolites and microbial communities was established by using Cytoscape software (version 3.9.0).

To predict the function of microbial communities, the PICRUSt software package was utilized. The evolutionary genetics of genes: Non-supervised Orthologous Groups database (eggNOG) and the Kyoto Encyclopedia of Genes and Genomes (KEGG) database were used for functional annotation.The statistical software SPSS (version 27.0) was used for variance analysis. The significance level was considered at *p* < 0.05.

## 3. Results and Discussion

### 3.1. Analysis of Physicochemical Indexes of Fermentation Broths

FMQ and IQ were applied to the fermentation experiment of Huangjiu, and the physicochemical indicators including total acid, total sugar, pH, and alcohol content during the fermentation process were monitored. The results are shown in Table 1.

According to Table 1, the total acid content ranged from 2.18 to 3.57 g/L during the brewing process of Huangjiu. In the wake of the fermentation progressing, an upward trend was reflected in the total acid content, which was consistent with the literature [24]. The total acid content of FMQFB was higher than that of IQFB. The total sugar content of fermentation mash was between 40.60 and 295.15 g/L. In the early stage of fermentation (1–5 days), the total sugar content of IQFB was slightly higher than that of FMQFB. Both of them showed an upward trend in the early stage, which reached the highest value on the fifth day. After adding water twice on the fifth day of brewing, the total sugar content decreased, and it still showed a downward trend in the later stage of fermentation. Moreover, the index of IQFB was lower than that of FMQFB in the later stage. When the fermentation was completed (27 days), the total sugar content in FMQFB and IQFB was 67.51 g/L and 40.60 g/L, respectively. The pH of Huangjiu fermented mash was between 3.62 and 3.98, and that of IQFB was a trifle higher than that of FMQFB. The alcohol content of FMQFB and IQFB on the 27th day of fermentation was 14.79% and 14.73%, respectively. The indicator of FMQFB was higher than that of IQFB in the early stage of fermentation, while IQFB had higher alcohol content than FMQFB in the middle stage. There was no difference in alcohol content at the end of fermentation. In the early stage of fermentation, the alcohol content increased rapidly and tended to be flat in the middle and late stages, which was consistent with the law of the fermentation process of millet Huangjiu [24].

### 3.2. Sequencing the Broth Fermented with Intensified Qu

PCR amplification and Illumina MiSeq sequencing were applied to analyze the diversity of bacterial and fungal communities during the fermentation process of IQFB. After eliminating low-quality reads and chimeras, 1,380,647 high-quality sequences with an average sequence length of 428 bp could be obtained in the bacterial community. On the basis of a similarity level of 97%, 230 operational classification units (OTUs), which were classified by the Silva classification database, were obtained through clustering using the algorithm named USEARCH11-uparse. In the fungal community, 1,395,768 high-quality sequences could be obtained with an average sequence length of 284 bp. The sequences of the fungi were clustered to obtain 77 OTUs, which were classified and annotated by the UNITE classification database. There was no prominent difference between the sequence numbers of bacteria and fungi in IQFB. Yet, the total number of bacterial OTUs was distinctly higher than that of fungal OTUs, which was consistent with the analysis of JIUYAO in Fangxian [25].

### 3.3. α-Diversity Analysis of Broth Fermented with Intensified Qu

**Figure 1:**
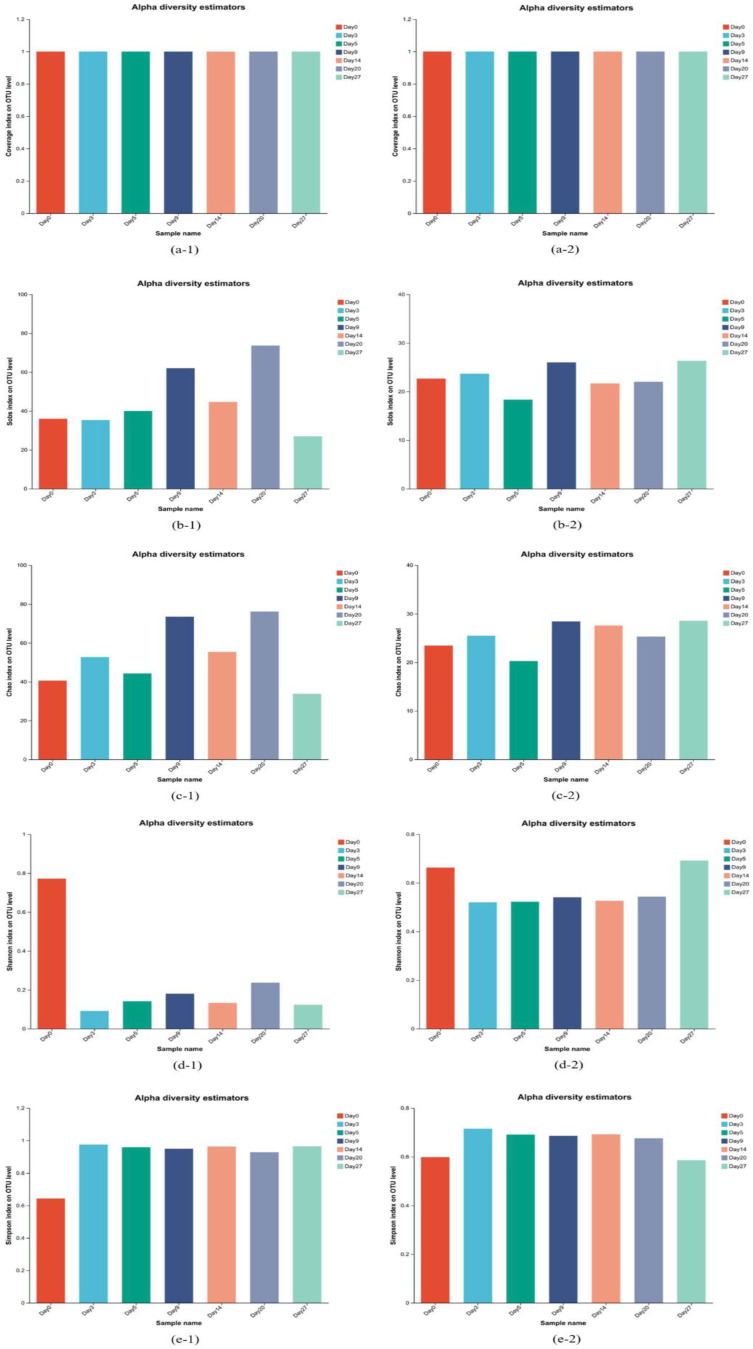
Distributions of alpha diversity estimators. ((**a-1**): Coverage diversity index of bacteria, (**a-2**): Coverage diversity index of fungi, (**b-1**): Sobs diversity index of bacteria, (**b-2**): Sobs diversity index of fungi, (**c-1**): Chao diversity index of bacteria, (**c-2**): Chao diversity index of fungi, (**d-1**): Shannon diversity index of bacteria, (**d-2**): Shannon diversity index of fungi, (**e-1**): Simpson diversity index of bacteria, (**e-2**): Simpson diversity index of fungi.).

The coverage, richness, and diversity of bacteria and fungi in different fermentation stages of IQFB were analyzed, and the distribution of Coverage, Sobs, Chao, Shannon, and Simpson as indices at the OUT level are displayed in Figure 1. The Coverage indices of bacteria and fungi in IQFB were greater than 99.90% and 99.99%, respectively, proving that the vast majority of sequences in the samples could be detected and could represent the true picture of microorganisms in IQFB. Higher Shannon and lower Simpson values symbolize higher community diversity. The diversity of bacterial communities was apparently higher in the blank sample without the addition of Qu than in the other fermentation broths. The fungal community had the highest diversity in the blank sample and IQFB on the 27th day. The maximum abundance and diversity of fungi in IQFB on the 27th day were probably due to the addition of two yeasts, *Wickerhamomyces anomalus* and *Saccharomyces cerevisiae*, to IQ. And the diversity index of fungi was lower than that of bacteria, which was consistent with the analysis results of OTUs.

### 3.4. Microbial Composition Analysis of Broth Fermented with Intensified Qu

**Figure 2:**
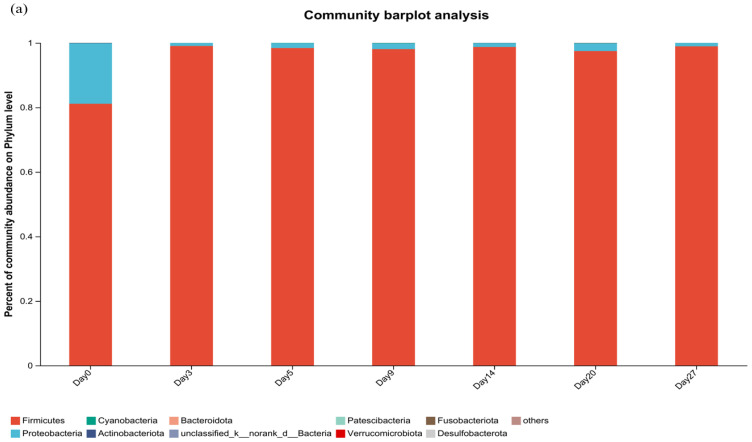

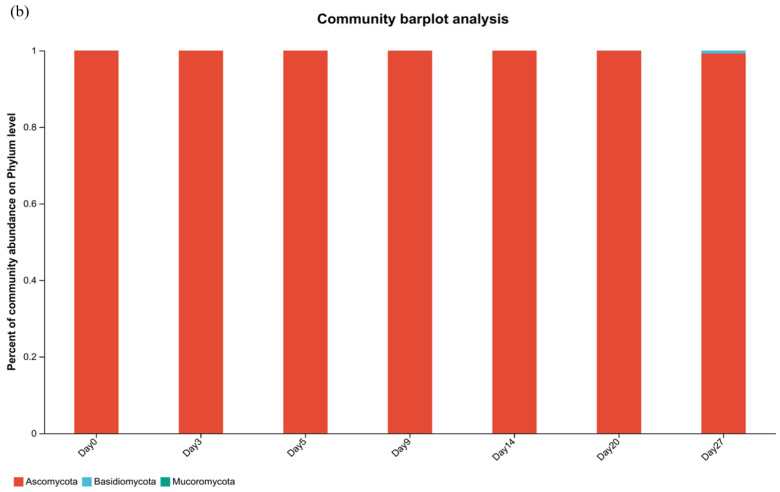
Community composition of phylum-level microorganisms in Huangjiu fermentation broths ((**a**) Bacterial relative abundance; (**b**) fungal relative abundance).

By sequencing, 20 phyla, 102 families, and 156 genera and 3 phyla, 40 families, and 54 genera were identified in the bacterial and fungal communities of IQFB. The top ranked species of bacteria at the phylum level including *Firmicutes*, *Proteobacteria*, *Cyanobacteria*, *Actinobacteriota*, and *Bacteroidota*, as presented in Figure 2a. However, only *Ascomycota*, *Basidiomycota*, and *Mucoromycota* phyla were identified for fungi, as shown in Figure 2b. In contrast to the previously reported sequencing results of FMQ in Fangxian [25], *unclassified_k_norank_d_bacteria* was also identified in the bacterial community, which also belonged to the dominant flora but accounted for a small percentage of IQFB. Furthermore, *Firmicutes* had a higher percentage in IQFB than in FMQ, greater than 97% in IQFB. In the fungal community, both demonstrated the same phylum, but Ascomycota had a higher percentage in IQFB, with all being greater than 99%.

**Figure 3:**
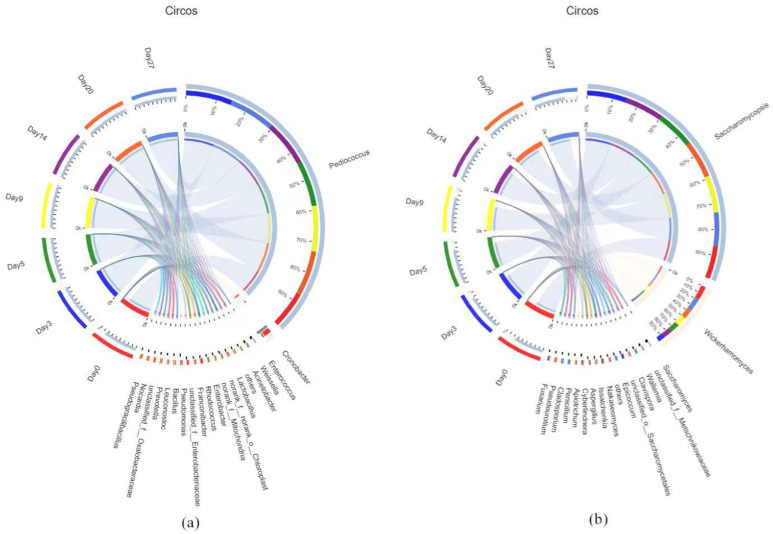
Circos samples and species at the genus level relationship diagrams ((**a**) Bacterial relative abundance; (**b**) fungal relative abundance).

The association between samples and species was explored to more visually analyze the percentage of species in different fermentation stages, and a Circos relationship diagram is shown in Figure 3. For the bacteria, *Pediococcus*, *Cronobacter*, *Enterococcus*, *Weissella*, and *Acinetobacter* were the prominent clusters. *Pediococcus* accounted for 79% of the blank sample, but this was far below its representation on day 3 (99%), day 5 (98%), day 9 (97%), day 14 (98%), day 20 (96%), and day 27 (98%). When the microbial community of Huangjiu produced around the Winter Solstice was evaluated, *Pediococcus* was recognized as the dominant genus in the later stages of fermentation [26]. It not only stimulates the fermentation of meat and vegetables [27], but also reduces the concentration of amines in the fermentation process [28]. *Pediococcus* generates acid but not gas, which encourages the production of some specialized flavor substances during the fermentation process [29]. There was a study revealed that *Weissella* and *Pediococcus* had a decisive role in the flavor characteristics of Huangjiu [30]. *Rhodococcus* was almost only present in the blank sample and on day 20. As for fungi, the prevailing phyla included *Saccharomycopsis*, *Wickerhamomyces*, and *Saccharomyces*, which constituted 99.70%, 99.48%, 99.39%, 99.60%, 99.37%, 99.41%, and 98.32% on day 0, day 3, day 5, day 9, day 14, day 20, and day 27, respectively. *Saccharomycopsis* was observed in 69% of the blank samples, while after the addition of Qu, its percentage was higher than 70% in all fermentation broths, with a maximum of 83% on day 3. *Saccharomycopsis* and *Wickerhamomyces* were also the dominant genera identified in FMQ, with *Wickerhamomyces* occupying more than 80% [25]. However, after adding 2% FMQ to IQ for Huangjiu fermentation, the percentage of bacterial genera changed noticeably, which may be associated with the environmental changes. Amylase, protease, and β-glucosidase could be secreted by *Saccharomycopsis*, which facilitates the glycation of starch [31]. *Saccharomycopsis* was identified as the main microbial community in JIUYAO of Shaoxing-jiu and exerted a vital influence on fermentation activity and flavor characteristics [32]. In the fermentation process of Huangjiu, *Saccharomyces* is one of the primary genera of yeasts that produce alcohol [33]. When *Saccharomycopsis* acted synergistically with *Wickerhamomyces*, it resulted in a significant contribution to the production of ethyl acetate as well as an improvement in the concentration of VACs such as *β*-phenethyl alcohol and phenethyl acetate [34]. Apart from this, *Wickerhamomyces* is one of the essential substances involved in the synthesis of phenethyl acetate [35].

### 3.5. Analysis of VACs in Huangjiu Fermentation Broths

By means of HS-SPME combined with GC-MS, VACs during the brewing process of FMQFB and IQFB were analyzed (Table 2). A total of 115 VACs were identified and quantified, including 28 esters, 27 alcohols, 18 acids, 13 aldehydes, 13 ketones, 5 furans, 1 sulfur compound, 2 nitrogen compounds, 4 phenols, and 4 hydrocarbons. Among them, a total of 49 VACs were detected in the blank fermentation broth, and 16 compounds such as 1-octen-3-ol were only present in the blank sample. There were 92 VACs discovered in FMQFB, with 29 compounds such as n-propanol only existing in FMQFB. Analogously, the Huangjiu brewed with IQ contained 74 kinds of VACs, of which 12 compounds, such as ethyl heptanoate, only occurred in IQFB.

**Figure 4:**
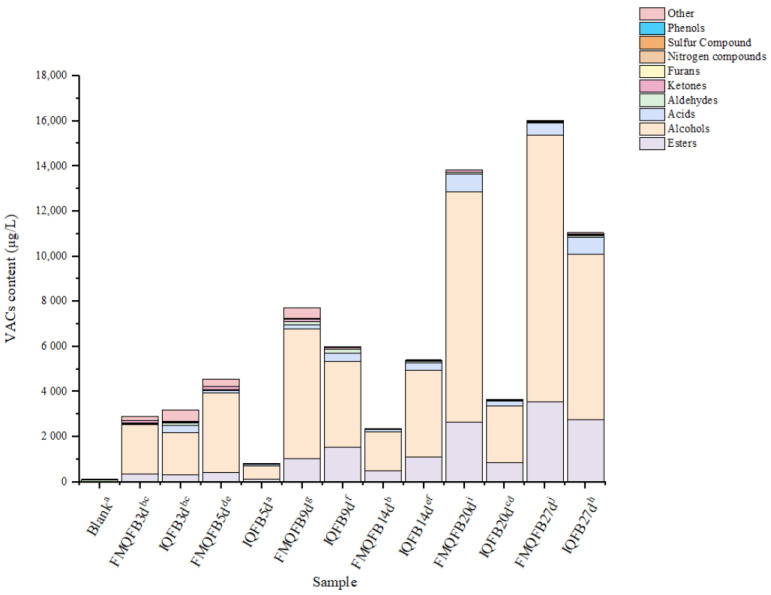
Contents of volatile flavor compounds in Huangjiu fermentation broths. Note: Different letters indicate significant differences in VACs content for both FMQFB and IQFB in relation to fermentation days.

The contents of VACs in Huangjiu fermentation broths are shown in Figure 4. From Figure 4, it can be seen that 117.74 µg/L of VACs were identified in the blank samples before brewing. Aldehydes were dominant, such as hexanal, octanal, and nonanal, which had higher concentrations and contributed to grain aroma [36]. Therefore, these aroma substances were mainly introduced from raw materials or produced during the cooking process. With the addition of Qu and fermentation, the quantities and content of VACs had a steep rise in Huangjiu on the 3rd day of fermentation, and a mass of liquid appeared. Through the determination of VACs in the fermentation broths after 3 days of fermentation, it was found that the VACs in FMQFB reached 2899.00 µg/L on the 3rd day, followed by a gradual increase in content. By the 14th day of fermentation, the content declined slightly, and then continued to increase. On the 27th day of fermentation, the content of VACs reached a maximum of 16,019.27 µg/L. The content of VACs in IQFB was 3199.93 µg/L on the 3rd day and decreased at high speed to 814.87 µg/L by the 5th day before continuing to increase. However, the concentrations decreased by about 30% on the 20th day, while they reached a maximum of 11,048.44 µg/L on the 27th day. Consistent with a previous report [24], the monitoring of VACs during the fermentation process revealed that their content was dynamically changing. Furthermore, the VACs of Huangjiu fermentation broth from different Qu were significantly different, which may be due to the various microbial communities of different Qu, and they had a notable impact on the characteristic aroma of the wine samples through synergistic growth [37]. In addition, during the course of fermentation, the content of aroma substances tended to present significant changes, which may be related to the interaction between microbial communities [1].

During the fermentation of FMQFB, the content of all VACs except aldehydes showed an increasing trend in the early stage of fermentation (1–5 d); in the middle stage (6–19 d), the content of aroma substances presented an up-trend followed by a small decrease and then continued to go up; in the late stage (20–27 d), the content of alcohols, esters, and furans exhibited a rising tendency, while other VACs showed a decreasing trend. In addition, during the fermentation of IQFB, an increasing trend followed by a decreasing trend was exhibited by most of the VACs in the early (1–5 d) and middle (6–19 d) periods; in the late fermentation period (20–27 d), a significant increase was presented by all VACs except nitrogenous and sulfurous compounds.

**Table 2:** Contents of volatile flavor compounds in fermented of Huangjiu.

Number	Volatile Aroma Compound	Content (µg/L)												
		Blank	FMQFB3d	FMQFB5d	FMQFB9d	FMQFB14d	FMQFB20d	FMQFB27d	IQFB3d	IQFB5d	IQFB9d	IQFB14d	IQFB20d	IQFB27d
1	Ethyl acetate	ND	ND	ND	ND	259.24 ± 48.41 ^a^	1103.20 ± 71.56 ^c^	1898.62 ± 163.69 ^d^	ND	59.89 ± 6.69 ^a^	1077.59 ± 110.46 ^c^	757.87 ± 89.04 ^b^	531.69 ± 53.22 ^b^	1938.47 ± 309.84 ^d^
2	Isoamyl acetate	ND	13.15 ± 2.01 ^a^	19.56 ± 2.80 ^a^	110.60 ± 17.44 ^cde^	38.08 ± 3.05 ^b^	97.22 ± 8.61 ^c^	123.08 ± 17.24 ^e^	44.60 ± 5.50 ^b^	8.94 ± 0.65 ^a^	145.12 ± 0.19 ^f^	117.41 ± 8.50 ^de^	102.28 ± 8.76 ^cd^	241.07 ± 19.56 ^g^
3	Isoamyl propanoate	ND	0.23 ± 0.04 ^a^	0.25 ± 0.03 ^a^	0.65 ± 0.10 ^b^	ND	ND	ND	ND	ND	ND	ND	ND	ND
4	Ethyl hexanoate	ND	1.81 ± 0.33 ^a^	2.44 ± 0.25 ^a^	15.57 ± 0.62 ^a^	ND	ND	ND	44.45 ± 5.66 ^b^	10.56 ± 1.32 ^a^	63.39 ± 5.69 ^c^	37.28 ± 5.13 ^b^	39.57 ± 0.81 ^b^	112.70 ± 20.08 ^d^
5	Ethyl heptanoate	ND	ND	ND	ND	ND	ND	ND	5.50 ± 0.53 ^c^	1.12 ± 0.06 ^a^	7.86 ± 1.56 ^d^	2.95 ± 0.43 ^b^	2.91 ± 0.18 ^b^	14.59 ± 1.31 ^e^
6	Ethyl lactate	ND	5.04 ± 0.47 ^a^	13.73 ± 2.09 ^a^	14.51 ± 2.55 ^a^	9.27 ± 1.27 ^a^	123.58 ± 23.40 ^b^	128.99 ± 22.89 ^b^	2.49 ± 0.30 ^a^	1.42 ± 0.02 ^a^	4.79 ± 0.74 ^a^	6.18 ± 0.61 ^a^	4.46 ± 0.69 ^a^	13.99 ± 2.23 ^a^
7	Hexyl formate	ND	7.24 ± 0.83	ND	ND	ND	ND	ND	ND	ND	ND	ND	ND	ND
8	Ethyl caprylate	ND	4.28 ± 0.51 ^a^	5.58 ± 0.65 ^a^	36.85 ± 2.99 ^c^	29.82 ± 4.61 ^bc^	225.39 ± 0.55 ^g^	189.95 ± 30.79 ^f^	47.91 ± 8.88 ^c^	12.35 ± 0.23 ^ab^	99.01 ± 7.72 ^d^	29.99 ± 1.70 ^bc^	35.19 ± 2.83 ^c^	126.50 ± 14.07 ^e^
9	Ethyl 3-hydroxybutyrate	ND	ND	ND	ND	ND	ND	ND	ND	ND	ND	ND	ND	1.59 ± 0.25
10	Ethyl nonanoate	ND	6.51 ± 0.99 ^d^	1.02 ± 0.04 ^a^	5.47 ± 0.45 ^cd^	3.62 ± 0.21 ^bc^	24.12 ± 2.23 ^g^	9.33 ± 1.03 ^e^	4.33 ± 0.85 ^bc^	ND	ND	2.77 ± 0.41 ^ab^	3.28 ± 0.59 ^bc^	16.55 ± 2.44 ^f^
11	Octyl formate	1.46 ± 0.25 ^a^	ND	ND	ND	3.02 ± 0.55 ^a^	ND	15.40 ± 0.90 ^c^	11.99 ± 2.29 ^b^	ND	ND	ND	ND	ND
12	Ethyl caprate	ND	5.33 ± 0.39 ^a^	8.20 ± 0.67 ^ab^	42.03 ± 3.63 ^c^	50.00 ± 8.56 ^c^	5.75 ± 0.34 ^a^	257.89 ± 21.83 ^d^	12.47 ± 2.33 ^ab^	4.34 ± 0.08 ^a^	15.50 ± 0.97 ^ab^	6.19 ± 1.21 ^a^	6.05 ± 0.42 ^a^	19.55 ± 3.66 ^b^
13	Ethyl (E)-4-decenoate	ND	ND	ND	ND	ND	ND	ND	3.83 ± 0.49 ^b^	0.78 ± 0.03 ^a^	8.03 ± 1.54 ^c^	2.08 ± 0.12 ^a^	1.48 ± 0.18 ^a^	ND
14	Diethyl succinate	ND	ND	ND	ND	3.05 ± 0.47 ^a^	10.30 ± 0.35 ^c^	10.38 ± 1.52 ^c^	ND	ND	ND	4.82 ± 0.33 ^b^	3.41 ± 0.23 ^ab^	11.36 ± 1.52 ^c^
15	Ethyl phenylacetate	ND	ND	4.57 ± 0.52 ^de^	8.46 ± 1.02 ^f^	2.40 ± 0.20 ^bc^	12.34 ± 2.36 ^g^	9.36 ± 0.55 ^f^	1.44 ± 0.07 ^ab^	0.50 ± 0.03 ^a^	4.05 ± 0.62 ^de^	3.11 ± 0.22 ^cd^	2.19 ± 0.19 ^bc^	5.22 ± 0.69 ^e^
16	Ethyl undecanoate	ND	ND	ND	ND	1.61 ± 0.16 ^a^	8.28 ± 0.28 ^c^	5.01 ± 0.94 ^b^	ND	ND	ND	ND	ND	ND
17	Methyl laurate	0.14 ± 0.02	ND	ND	ND	ND	ND	ND	ND	ND	ND	ND	ND	ND
18	Phenethyl acetate	0.40 ± 0.05 ^a^	74.32 ± 8.30 ^d^	81.02 ± 12.07 ^d^	207.55 ± 18.58 ^g^	45.85 ± 8.17 ^c^	235.38 ± 5.98 ^h^	141.22 ± 20.28 ^f^	24.73 ± 4.56 ^b^	8.48 ± 0.07 ^ab^	71.92 ± 5.57 ^d^	73.60 ± 3.07 ^d^	47.15 ± 7.52 ^c^	105.27 ± 13.51 ^e^
19	Ethyl laurate	ND	6.12 ± 1.08 ^bcd^	5.30 ± 0.91 ^bc^	30.42 ± 0.71 ^e^	9.22 ± 1.20 ^d^	113.54 ± 5.14 ^g^	56.53 ± 3.39 ^f^	3.42 ± 0.34 ^ab^	1.10 ± 0.05 ^a^	3.79 ± 0.12 ^ab^	3.98 ± 0.11 ^ab^	3.70 ± 0.61 ^ab^	8.07 ± 1.3 ^cd^
20	Phenethyl isobutyrate	ND	ND	ND	ND	1.28 ± 0.22 ^a^	9.66 ± 0.89 ^b^	9.96 ± 0.99 ^b^	ND	ND	ND	ND	ND	ND
21	Isopropyl myristate	0.17 ± 0.03 ^a^	3.56 ± 0.53 ^f^	3.76 ± 0.74 ^f^	2.39 ± 0.41 ^cd^	1.96 ± 0.33 ^bc^	1.22 ± 0.22 ^b^	3.44 ± 0.14 ^ef^	1.83 ± 0.31 ^bc^	1.09 ± 0.12 ^b^	2.94 ± 0.47 ^def^	2.88 ± 0.38 ^def^	2.59 ± 0.47 ^cde^	6.86 ± 1.23 ^g^
22	Ethyl tetradecanoate	0.33 ± 0.04 ^a^	59.98 ± 9.95 ^d^	70.81 ± 9.01 ^e^	170.33 ± 16.04 ^h^	6.78 ± 1.29 ^ab^	124.78 ± 8.37 ^g^	109.95 ± 1.98 ^f^	16.95 ± 2.35 ^bc^	5.93 ± 0.41 ^ab^	5.45 ± 0.17 ^ab^	7.03 ± 1.30 ^ab^	7.39 ± 0.82 ^ab^	22.13 ± 2.48 ^c^
23	Ethyl pentadecanoate	ND	1.17 ± 0.18 ^a^	2.16 ± 0.17 ^a^	12.92 ± 2.43 ^c^	0.54 ± 0.06 ^a^	5.82 ± 1.78 ^b^	2.90 ± 0.25 ^a^	ND	ND	ND	ND	ND	ND
24	Methyl hexadecanoate	0.13 ± 0.03 ^a^	2.71 ± 0.28 ^g^	2.94 ± 0.40 ^gh^	3.11 ± 0.23 ^h^	0.26 ± 0.04 ^ab^	ND	1.02 ± 0.18 ^de^	1.90 ± 0.31 ^f^	0.52 ± 0.04 ^bc^	0.93 ± 0.06 ^de^	0.70 ± 0.11 ^cd^	0.42 ± 0.02 ^abc^	1.17 ± 0.15 ^e^
25	Ethyl hexadecanoate	2.26 ± 0.39 ^a^	124.49 ± 20.21 ^d^	163.50 ± 14.90 ^e^	304.92 ± 49.83 ^f^	22.84 ± 4.17 ^ab^	415.29 ± 42.15 ^g^	438.39 ± 9.62 ^g^	44.67 ± 4.48 ^b^	14.49 ± 0.94 ^ab^	16.80 ± 1.45 ^ab^	23.03 ± 2.51 ^ab^	24.29 ± 0.32 ^ab^	79.87 ± 6.03 ^c^
26	Ethyl octadecanoate	0.08 ± 0.01 ^a^	1.80 ± 0.27 ^b^	3.91 ± 0.37 ^c^	7.12 ± 1.35 ^d^	1.11 ± 0.06 ^ab^	14.32 ± 1.93 ^f^	11.00 ± 0.60 ^e^	0.50 ± 0.09 ^a^	0.15 ± 0.02 ^a^	0.47 ± 0.09 ^a^	0.40 ± 0.04 ^a^	0.37 ± 0.01 ^a^	1.08 ± 0.07 ^ab^
27	Ethyl oleate	0.28 ± 0.04 ^a^	11.68 ± 2.00 ^b^	19.27 ± 1.72 ^c^	32.25 ± 4.96 ^d^	4.34 ± 0.51 ^a^	55.00 ± 5.22 ^e^	60.08 ± 3.99 ^f^	8.64 ± 1.35 ^b^	2.87 ± 0.09 ^a^	2.07 ± 0.22 ^a^	2.90 ± 0.27 ^a^	3.55 ± 0.03 ^a^	10.38 ± 0.65 ^b^
28	Ethyl linoleate	ND	9.99 ± 1.08 ^c^	14.11 ± 0.73 ^d^	27.76 ± 2.34 ^e^	4.33 ± 0.57 ^ab^	71.46 ± 3.29 ^f^	75.55 ± 2.74 ^g^	10.40 ± 1.94 ^c^	4.46 ± 0.81 ^ab^	2.99 ± 0.35 ^a^	2.04 ± 0.25 ^a^	6.64 ± 0.02 ^b^	15.92 ± 2.21 ^d^
29	1-Propanol	ND	14.40 ± 1.97 ^a^	23.14 ± 2.93 ^b^	33.22 ± 4.47 ^c^	ND	ND	ND	ND	ND	ND	ND	ND	ND
30	Isobutanol	ND	121.68 ± 19.03 ^b^	199.91 ± 27.18 ^c^	305.69 ± 25.41 ^d^	145.26 ± 25.88 ^bc^	694.84 ± 74.55 ^f^	1104.95 ± 36.40 ^h^	148.34 ± 16.10 ^bc^	48.54 ± 0.53 ^a^	414.38 ± 22.41 ^e^	385.89 ± 24.65 ^e^	210.95 ± 0.74 ^c^	807.99 ± 75.19 ^g^
31	1-Butanol	ND	ND	3.78 ± 0.26 ^a^	6.13 ± 0.94 ^ab^	4.73 ± 0.49 ^ab^	25.03 ± 2.55 ^e^	34.32 ± 4.31 ^f^	ND	ND	8.20 ± 1.37 ^bc^	9.89 ± 1.28 ^c^	3.73 ± 0.47 ^a^	16.53 ± 2.84 ^d^
32	Isoamylol	ND	759.20 ± 72.89 ^ab^	1109.64 ± 163.39 ^b^	2107.23 ± 348.28 ^d^	870.47 ± 65.35 ^ab^	5255.80 ± 576.22 ^f^	5907.94 ± 853.37 ^g^	887.88 ± 141.12 ^ab^	240.26 ± 0.57 ^a^	1889.98 ± 181.48 ^cd^	2023.15 ± 294.59 ^d^	1312.34 ± 114.59 ^bc^	3890.74 ± 468.85 ^e^
33	1-Pentanol	ND	0.95 ± 0.12 ^a^	1.54 ± 0.25 ^b^	1.42 ± 0.17 ^b^	ND	ND	ND	ND	ND	ND	ND	ND	ND
34	2-Heptanol	ND	0.58 ± 0.04 ^b^	0.44 ± 0.05 ^a^	0.60 ± 0.04 ^b^	ND	ND	ND	ND	ND	ND	ND	ND	ND
35	1-Hexanol	0.89 ± 0.14 ^a^	ND	9.13 ± 1.42 ^c^	9.12 ± 0.71 ^c^	5.59 ± 0.52 ^b^	15.82 ± 2.45 ^d^	24.26 ± 0.58 ^f^	15.78 ± 1.13 ^d^	4.18 ± 0.06 ^b^	19.29 ± 3.04 ^e^	17.32 ± 3.43 ^de^	11.48 ± 2.16 ^c^	23.23 ± 3.34 ^f^
36	1-Octen-3-ol	4.83 ± 0.73	ND	ND	ND	ND	ND	ND	ND	ND	ND	ND	ND	ND
37	1-Heptanol	0.56 ± 0.07	ND	ND	ND	ND	ND	ND	ND	ND	ND	ND	ND	ND
38	2-Ethylhexanol	1.81 ± 0.31 ^a^	4.49 ± 0.53 ^b^	3.27 ± 0.54 ^ab^	12.52 ± 2.39 ^f^	2.53 ± 0.28 ^a^	8.99 ± 1.25 ^de^	8.43 ± 1.11 ^de^	6.46 ± 0.63 ^c^	2.95 ± 0.07 ^ab^	7.41 ± 0.46 ^cd^	8.16 ± 0.32 ^cde^	7.40 ± 1.01 ^cd^	9.79 ± 1.56 ^e^
39	2,3-Butanediol	0.46 ± 0.07 ^a^	22.62 ± 3.99 ^ab^	48.61 ± 7.68 ^abc^	25.70 ± 4.22 ^ab^	82.99 ± 14.73 ^abc^	783.23 ± 55.80 ^e^	873.24 ± 146.08 ^f^	101.80 ± 11.81 ^bc^	22.32 ± 0.13 ^ab^	124.32 ± 17.38 ^c^	93.88 ± 6.88 ^bc^	75.00 ± 7.76 ^abc^	391.02 ± 31.91 ^d^
40	1-Octanol	ND	10.83 ± 1.61 ^bc^	12.28 ± 2.42 ^c^	12.79 ± 2.01 ^c^	ND	16.64 ± 1.83 ^d^	ND	ND	4.43 ± 0.09 ^a^	13.32 ± 1.19 ^c^	11.49 ± 0.60 ^bc^	9.16 ± 1.11 ^b^	17.61 ± 2.43 ^d^
41	1,2-Propanediol	ND	ND	ND	ND	ND	31.82 ± 2.63 ^d^	16.83 ± 2.64 ^c^	2.24 ± 0.38 ^a^	0.36 ± 0.07 ^a^	2.98 ± 0.37 ^a^	2.36 ± 0.43 ^a^	2.07 ± 0.08 ^a^	8.60 ± 0.97 ^b^
42	1-Nonanol	ND	5.32 ± 0.65 ^bc^	4.14 ± 0.69 ^ab^	9.18 ± 1.67 ^def^	3.01 ± 0.09 ^ab^	14.53 ± 0.16 ^h^	11.75 ± 1.66 ^g^	7.35 ± 0.84 ^cd^	2.61 ± 0.04 ^a^	10.64 ± 0.90 ^fg^	9.90 ± 0.51 ^efg^	7.58 ± 0.78 ^cde^	19.53 ± 3.73 ^i^
43	Methionol	ND	2.99 ± 0.55 ^abc^	4.47 ± 0.81 ^c^	4.42 ± 0.61 ^c^	1.75 ± 0.20 ^a^	14.52 ± 1.69 ^e^	16.03 ± 2.63 ^e^	4.18 ± 0.20 ^bc^	2.70 ± 0.32 ^abc^	4.06 ± 0.54 ^bc^	3.50 ± 0.17 ^abc^	2.40 ± 0.42 ^ab^	6.27 ± 0.86 ^d^
44	1-Decanol	ND	4.33 ± 0.81 ^a^	37.68 ± 3.28 ^b^	6.68 ± 0.61 ^a^	ND	ND	ND	ND	ND	ND	ND	ND	ND
45	Citronellol	ND	2.25 ± 0.33 ^a^	ND	54.64 ± 8.56 ^c^	2.32 ± 0.03 ^a^	16.41 ± 2.02 ^b^	14.49 ± 1.66 ^b^	ND	ND	ND	4.85 ± 0.44 ^a^	3.21 ± 0.55 ^a^	7.29 ± 1.13 ^a^
46	Nerol	ND	ND	ND	ND	ND	ND	ND	0.77 ± 0.15 ^b^	0.28 ± 0.03 ^a^	1.62 ± 0.08 ^d^	1.33 ± 0.09 ^c^	0.85 ± 0.10 ^b^	1.90 ± 0.18 ^e^
47	Benzyl alcohol	ND	ND	ND	ND	ND	2.61 ± 0.03	ND	ND	ND	ND	ND	ND	ND
48	Phenethyl alcohol	4.52 ± 0.71 ^a^	1215.15 ± 237.26 ^c^	2034.77 ± 297.88 ^d^	3093.32 ± 152.50 ^e^	583.96 ± 47.78 ^b^	3272.66 ± 29.69 ^e^	3788.04 ± 453.37 ^f^	694.05 ± 108.53 ^b^	223.08 ± 2.72 ^a^	1307.90 ± 81.94 ^c^	1256.98 ± 33.68 ^c^	857.64 ± 128.43 ^b^	2123.31 ± 339.58 ^d^
49	1-Dodecanol	0.07 ± 0.01 ^a^	8.11 ± 1.54 ^c^	9.59 ± 1.19 ^cd^	24.44 ± 2.43 ^h^	4.33 ± 0.30 ^b^	18.62 ± 0.58 ^g^	12.86 ± 1.11 ^ef^	4.39 ± 0.62 ^b^	3.21 ± 0.31 ^b^	11.76 ± 1.56 ^e^	11.08 ± 0.42 ^de^	8.58 ± 0.70 ^c^	13.76 ± 1.31 ^f^
50	Cedrol	ND	ND	ND	ND	0.77 ± 0.07 ^a^	2.79 ± 0.33 ^d^	ND	ND	ND	ND	2.02 ± 0.11 ^c^	1.49 ± 0.20 ^b^	1.92 ± 0.31 ^c^
51	*β*-Eudesmol	ND	1.31 ± 0.25 ^a^	1.62 ± 0.17 ^a^	2.81 ± 0.31 ^b^	ND	ND	ND	ND	ND	ND	ND	ND	ND
52	1-Tetradecanol	ND	4.83 ± 0.93 ^b^	4.56 ± 0.51 ^b^	5.75 ± 0.41 ^c^	0.46 ± 0.05 ^a^	4.17 ± 0.96 ^b^	ND	ND	ND	ND	ND	ND	ND
53	(2E,6E)-Farnesol	ND	2.11 ± 0.32 ^bc^	2.45 ± 0.46 ^c^	4.15 ± 0.36 ^d^	0.55 ± 0.06 ^a^	13.98 ± 1.85 ^e^	2.88 ± 0.35 ^c^	ND	0.20 ± 0.03 ^a^	1.28 ± 0.07 ^ab^	0.82 ± 0.10 ^a^	0.51 ± 0.06 ^a^	1.08 ± 0.09 ^ab^
54	1-Hexadecanol	0.94 ± 0.15 ^a^	4.87 ± 0.78 ^cd^	7.33 ± 0.91 ^e^	7.49 ± 0.95 ^e^	2.71 ± 0.45 ^b^	7.04 ± 1.26 ^e^	5.75 ± 0.99 ^d^	4.65 ± 0.63 ^cd^	1.45 ± 0.09 ^a^	5.64 ± 0.46 ^d^	3.64 ± 0.23 ^bc^	2.65 ± 0.45 ^b^	5.66 ± 0.75 ^d^
55	4-Hydroxyphenethyl alcohol	ND	ND	ND	5.60 ± 0.98 ^d^	0.97 ± 0.14 ^ab^	ND	ND	0.53 ± 0.03 ^a^	0.17 ± 0.03 ^a^	1.59 ± 0.27 ^bc^	2.30 ± 0.34 ^c^	1.77 ± 0.26 ^bc^	6.61 ± 0.72 ^e^
56	Acetic acid	ND	22.03 ± 3.31 ^a^	51.20 ± 8.88 ^a^	68.46 ± 4.06 ^a^	71.21 ± 7.49 ^a^	707.39 ± 101.81 ^e^	435.98 ± 74.23 ^c^	257.57 ± 38.51 ^b^	54.72 ± 5.95 ^a^	230.66 ± 42.64 ^b^	254.78 ± 18.40 ^b^	173.73 ± 26.90 ^b^	598.69 ± 107.08 ^d^
57	Isobutyric acid	ND	ND	5.89 ± 0.80 ^a^	6.38 ± 1.16 ^a^	6.42 ± 0.82 ^a^	28.14 ± 2.33 ^e^	28.72 ± 2.50 ^e^	13.83 ± 2.46 ^bc^	4.40 ± 0.18 ^a^	19.77 ± 3.87 ^d^	17.71 ± 2.45 ^cd^	10.73 ± 1.28 ^b^	30.58 ± 4.28 ^e^
58	Butyric acid	ND	1.53 ± 0.26 ^a^	2.02 ± 0.32 ^a^	ND	ND	ND	ND	ND	ND	ND	ND	ND	ND
59	2-Methylbutyric acid	ND	2.38 ± 0.45 ^a^	2.41 ± 0.34 ^a^	5.97 ± 0.81 ^b^	3.38 ± 0.57 ^ab^	15.37 ± 3.51 ^d^	11.45 ± 1.72 ^c^	ND	ND	ND	ND	ND	ND
60	2-Methylhexanoic acid	ND	ND	ND	ND	ND	ND	ND	7.34 ± 0.52 ^b^	2.22 ± 0.07 ^a^	9.45 ± 1.18 ^c^	9.55 ± 1.36 ^c^	5.99 ± 0.64 ^b^	14.20 ± 1.14 ^d^
61	Hexanoic acid	0.30 ± 0.04 ^a^	ND	2.52 ± 0.14 ^b^	2.80 ± 0.34 ^bc^	ND	ND	4.48 ± 0.69 ^c^	12.18 ± 2.31 ^f^	2.86 ± 0.10 ^bc^	9.39 ± 1.59 ^e^	7.26 ± 0.54 ^d^	4.71 ± 0.70 ^c^	8.41 ± 1.41 ^de^
62	Octanoic acid	0.11 ± 0.02 ^a^	5.79 ± 0.87 ^cd^	7.80 ± 0.79 ^cde^	33.04 ± 4.73 ^h^	5.63 ± 0.97 ^c^	31.44 ± 1.61 ^h^	17.15 ± 2.18 ^g^	9.15 ± 1.38 ^ef^	2.11 ± 0.24 ^ab^	11.92 ± 1.54 ^f^	7.48 ± 0.58 ^cde^	4.91 ± 0.86 ^bc^	8.87 ± 0.84 ^de^
63	Nonanoic acid	0.14 ± 0.03 ^a^	5.02 ± 0.43 ^a^	7.22 ± 1.24 ^ab^	12.20 ± 1.65 ^b^	2.94 ± 0.20 ^a^	3.86 ± 0.71 ^a^	33.22 ± 4.73 ^d^	23.14 ± 2.88 ^c^	6.63 ± 1.16 ^ab^	39.78 ± 7.49 ^d^	25.28 ± 2.56 ^c^	20.73 ± 2.58 ^c^	62.83 ± 9.82 ^e^
64	Decanoic acid	ND	ND	ND	25.37 ± 1.26 ^g^	2.73 ± 0.22 ^ab^	20.42 ± 0.73 ^f^	9.55 ± 1.14 ^e^	4.48 ± 0.49 ^cd^	1.50 ± 0.14 ^a^	5.47 ± 0.78 ^d^	4.62 ± 0.10 ^cd^	3.69 ± 0.03 ^bc^	9.54 ± 1.69 ^e^
65	Levulinic acid	ND	ND	ND	ND	ND	ND	ND	ND	0.49 ± 0.03 ^a^	2.17 ± 0.09 ^c^	1.09 ± 0.17 ^b^	0.81 ± 0.04 ^ab^	2.67 ± 0.42 ^d^
66	Benzoic acid	ND	0.78 ± 0.11 ^a^	1.66 ± 0.25 ^b^	ND	ND	ND	ND	ND	ND	ND	ND	ND	ND
67	Lauric acid	ND	3.21 ± 0.32 ^c^	4.93 ± 0.48 ^d^	5.42 ± 0.54 ^d^	0.42 ± 0.06 ^a^	ND	ND	ND	0.54 ± 0.05 ^a^	1.36 ± 0.09 ^b^	1.30 ± 0.09 ^b^	0.90 ± 0.04 ^ab^	2.88 ± 0.55 ^c^
68	Myristic acid	ND	5.37 ± 0.68 ^a^	6.91 ± 0.89 ^a^	13.22 ± 1.84 ^b^	ND	ND	ND	ND	ND	ND	ND	ND	ND
69	Palmitic acid	0.28 ± 0.06 ^a^	9.27 ± 1.16 ^b^	14.44 ± 2.18 ^c^	26.67 ± 4.37 ^d^	0.51 ± 0.07 ^a^	ND	ND	0.86 ± 0.11 ^a^	0.18 ± 0.03 ^a^	0.98 ± 0.12 ^a^	0.92 ± 0.04 ^a^	0.49 ± 0.06 ^a^	2.38 ± 0.44 ^a^
70	Palmitoleic acid	ND	ND	ND	2.72 ± 0.39	ND	ND	ND	ND	ND	ND	ND	ND	ND
71	Stearic acid	ND	0.39 ± 0.03 ^a^	2.79 ± 0.08 ^b^	3.84 ± 0.50 ^c^	ND	ND	ND	ND	ND	ND	ND	ND	ND
72	Oleic acid	ND	ND	0.68 ± 0.12 ^a^	1.09 ± 0.20 ^b^	ND	ND	ND	ND	ND	ND	ND	ND	ND
73	Linoleic acid	ND	ND	0.19 ± 0.01	ND	ND	ND	ND	ND	ND	ND	ND	ND	ND
74	Acetaldehyde	ND	ND	ND	ND	ND	ND	ND	83.98 ± 15.82 ^d^	10.77 ± 0.33 ^a^	129.47 ± 9.44 ^e^	48.66 ± 6.80 ^c^	25.43 ± 2.69 ^b^	56.04 ± 2.82 ^c^
75	Hexanal	50.60 ± 6.13 ^b^	ND	ND	ND	ND	ND	ND	5.67 ± 0.67 ^a^	ND	ND	ND	ND	ND
76	Heptanal	2.95 ± 0.42	ND	ND	ND	ND	ND	ND	ND	ND	ND	ND	ND	ND
77	Octanal	4.07 ± 0.30	ND	ND	ND	ND	ND	ND	ND	ND	ND	ND	ND	ND
78	trans-2-Heptenal	1.87 ± 0.29	ND	ND	ND	ND	ND	ND	ND	ND	ND	ND	ND	ND
79	Nonanal	8.16 ± 0.46 ^c^	10.58 ± 1.57 ^de^	8.35 ± 0.98 ^cd^	16.05 ± 2.56 ^f^	1.75 ± 0.16 ^a^	6.26 ± 0.95 ^bc^	8.51 ± 0.91 ^cd^	5.06 ± 1.00 ^b^	11.23 ± 1.94 ^e^	7.74 ± 1.04 ^c^	8.37 ± 0.77 ^cd^	6.83 ± 0.41 ^bc^	11.16 ± 1.25 ^e^
80	Decanal	1.90 ± 0.28 ^bc^	1.21 ± 0.22 ^a^	ND	ND	ND	2.17 ± 0.28 ^c^	1.44 ± 0.20 ^ab^	ND	ND	ND	ND	ND	3.41 ± 0.55 ^d^
81	Benzaldehyde	1.88 ± 0.22 ^a^	31.09 ± 4.33 ^d^	10.06 ± 1.42 ^ab^	93.42 ± 15.48 ^g^	11.20 ± 1.31 ^ab^	39.62 ± 3.69 ^e^	17.46 ± 2.25 ^bc^	7.50 ± 0.45 ^a^	2.47 ± 0.04 ^a^	61.11 ± 3.46 ^f^	23.14 ± 4.16 ^cd^	8.59 ± 0.86 ^ab^	10.86 ± 1.58 ^ab^
82	(2E)-2-Nonenal	1.10 ± 0.12	ND	ND	ND	ND	ND	ND	ND	ND	ND	ND	ND	ND
83	(2E)-2-Decenal	0.86 ± 0.12	ND	ND	ND	ND	ND	ND	ND	ND	ND	ND	ND	ND
84	trans-2-Undecenal	0.34 ± 0.02 ^a^	0.64 ± 0.04 ^ab^	0.50 ± 0.05 ^a^	1.24 ± 0.19 ^d^	ND	ND	ND	ND	ND	ND	1.16 ± 0.09 ^cd^	0.92 ± 0.01 ^bc^	2.10 ± 0.40 ^e^
85	Dodecanal	0.47 ± 0.04 ^a^	ND	ND	ND	0.63 ± 0.05 ^ab^	ND	2.50 ± 0.42 ^d^	0.81 ± 0.08 ^ab^	2.13 ± 0.35 ^cd^	1.96 ± 0.19 ^c^	1.82 ± 0.28 ^c^	1.03 ± 0.09 ^b^	1.79 ± 0.27 ^c^
86	4-Hydroxybenzaldehyde	ND	ND	ND	1.49 ± 0.24	ND	ND	ND	ND	ND	ND	ND	ND	ND
87	2-Heptanone	ND	ND	ND	0.30 ± 0.04	ND	ND	ND	ND	ND	ND	ND	ND	ND
88	2-Octanone	0.98 ± 0.17	ND	ND	ND	ND	ND	ND	ND	ND	ND	ND	ND	ND
89	Acetoin	ND	82.56 ± 4.91 ^c^	143.66 ± 18.15 ^e^	114.18 ± 15.66 ^d^	31.74 ± 2.74 ^b^	103.51 ± 19.01 ^d^	32.54 ± 1.07 ^b^	38.08 ± 6.55 ^b^	4.44 ± 0.19 ^a^	46.09 ± 7.96 ^b^	8.76 ± 0.83 ^a^	2.44 ± 0.06 ^a^	3.99 ± 0.76 ^a^
90	2,3-Octanedione	1.18 ± 0.10	ND	ND	ND	ND	ND	ND	ND	ND	ND	ND	ND	ND
91	6-Methylhept-5-en-2-one	0.45 ± 0.03 ^a^	ND	ND	1.90 ± 0.18 ^b^	ND	ND	ND	ND	ND	ND	ND	ND	ND
92	2-Nonanone	ND	ND	ND	1.71 ± 0.09	ND	ND	ND	ND	ND	ND	ND	ND	ND
93	3-Nonen-2-one	0.43 ± 0.06	ND	ND	ND	ND	ND	ND	ND	ND	ND	ND	ND	ND
94	2-Undecanone	0.42 ± 0.08	ND	ND	ND	ND	ND	ND	ND	ND	ND	ND	ND	ND
95	Acetophenone	ND	3.83 ± 0.76 ^d^	6.50 ± 0.92 ^e^	ND	ND	ND	5.90 ± 0.79 ^e^	2.04 ± 0.11 ^bc^	0.56 ± 0.02 ^a^	1.98 ± 0.21 ^bc^	1.89 ± 0.22 ^bc^	1.20 ± 0.08 ^ab^	2.79 ± 0.29 ^c^
96	4-Hydroxy-2-butanone	ND	ND	ND	ND	ND	ND	ND	ND	0.47 ± 0.04 ^a^	2.37 ± 0.18 ^d^	1.82 ± 0.14 ^c^	1.23 ± 0.16 ^b^	2.85 ± 0.41 ^e^
97	Neryl acetone	1.16 ± 0.16 ^abc^	3.23 ± 0.56 ^d^	1.96 ± 0.12 ^c^	8.90 ± 1.17 ^e^	ND	ND	ND	0.53 ± 0.08 ^ab^	0.37 ± 0.04 ^a^	1.69 ± 0.29 ^c^	1.81 ± 0.06 ^c^	1.24 ± 0.16 ^bc^	3.04 ± 0.58 ^d^
98	2-Pentadecanone	0.69 ± 0.10 ^bc^	2.51 ± 0.16 ^g^	ND	ND	ND	1.76 ± 0.13 ^f^	1.38 ± 0.14 ^e^	1.02 ± 0.12 ^d^	0.34 ± 0.04 ^a^	0.79 ± 0.05 ^c^	0.52 ± 0.03 ^ab^	0.42 ± 0.08 ^a^	1.04 ± 0.18 ^d^
99	Phytone	0.22 ± 0.03 ^a^	2.39 ± 0.42 ^d^	2.52 ± 0.43 ^d^	ND	ND	1.86 ± 0.11 ^c^	ND	0.87 ± 0.13 ^b^	0.38 ± 0.07 ^a^	1.06 ± 0.13 ^b^	0.83 ± 0.07 ^b^	0.79 ± 0.13 ^b^	1.82 ± 0.29 ^c^
100	2-Butylfuran	0.92 ± 0.14	ND	ND	ND	ND	ND	ND	ND	ND	ND	ND	ND	ND
101	2-Pentylfuran	13.14 ± 2.29	ND	ND	ND	ND	ND	ND	ND	ND	ND	ND	ND	ND
102	*γ*-Butyrolactone	ND	ND	1.68 ± 0.26 ^a^	7.71 ± 0.93 ^c^	3.25 ± 0.59 ^ab^	12.81 ± 1.84 ^d^	19.03 ± 2.96 ^e^	2.75 ± 0.43 ^a^	1.35 ± 0.06 ^a^	4.99 ± 0.18 ^b^	5.27 ± 0.29 ^b^	2.04 ± 0.33 ^a^	14.51 ± 1.82 ^d^
103	*γ*-Nonanolactone	0.28 ± 0.03 ^a^	ND	3.47 ± 0.69 ^bc^	5.18 ± 0.31 ^c^	2.70 ± 0.49 ^b^	4.51 ± 0.42 ^c^	4.37 ± 0.65 ^bc^	11.89 ± 1.83 ^ef^	3.64 ± 0.09 ^bc^	13.22 ± 0.83 ^f^	11.35 ± 0.94 ^e^	7.58 ± 1.37 ^d^	16.07 ± 2.00 ^g^
104	(Z)-dihydro-5-(2-octenyl)furan-2(3H)-one	0.05 ± 0.01 ^a^	1.80 ± 0.27 ^b^	ND	ND	ND	ND	ND	ND	ND	ND	ND	ND	ND
105	2-Acetylpyrazine	ND	1.27 ± 0.24 ^a^	1.38 ± 0.22 ^a^	ND	ND	ND	ND	ND	ND	ND	ND	ND	ND
106	Indole	0.73 ± 0.03 ^d^	0.21 ± 0.03 ^b^	0.89 ± 0.13 ^e^	ND	ND	ND	ND	0.37 ± 0.03 ^c^	0.06 ± 0.01 ^a^	0.91 ± 0.08 ^e^	0.29 ± 0.05 ^bc^	0.08 ± 0.01 ^a^	ND
107	4-Methyl-5-vinylthiazole	ND	ND	0.96 ± 0.18	ND	ND	ND	ND	ND	ND	ND	ND	ND	ND
108	Phenol	0.26 ± 0.04 ^a^	1.33 ± 0.25 ^b^	ND	1.85 ± 0.20 ^cd^	1.83 ± 0.35 ^cd^	2.59 ± 0.22 ^e^	2.25 ± 0.40 ^de^	1.73 ± 0.33 ^bc^	0.68 ± 0.07 ^a^	2.59 ± 0.29 ^e^	2.21 ± 0.15 ^de^	1.63 ± 0.23 ^bc^	3.81 ± 0.32 ^f^
109	4-Ethyl guaiacol	ND	ND	ND	ND	0.24 ± 0.04 ^a^	ND	ND	8.15 ± 0.99 ^e^	2.33 ± 0.09 ^b^	8.08 ± 0.71 ^e^	6.26 ± 0.45 ^d^	4.03 ± 0.55 ^c^	8.74 ± 1.19 ^e^
110	4-Ethylphenol	ND	ND	ND	ND	ND	ND	ND	3.16 ± 0.54 ^c^	0.84 ± 0.08 ^a^	2.98 ± 0.08 ^c^	2.14 ± 0.19 ^b^	1.28 ± 0.13 ^a^	3.29 ± 0.32 ^c^
111	2-Methoxy-4-vinylphenol	0.42 ± 0.07 ^a^	4.08 ± 0.19 ^c^	6.63 ± 0.64 ^e^	6.92 ± 0.80 ^e^	1.62 ± 0.31 ^b^	5.40 ± 0.55 ^d^	3.52 ± 0.66 ^c^	4.10 ± 0.32 ^c^	1.43 ± 0.10 ^b^	5.09 ± 0.37 ^d^	3.75 ± 0.63 ^c^	2.17 ± 0.23 ^b^	6.39 ± 0.77 ^e^
112	1,1-Diethoxyethane	1.89 ± 0.15 ^a^	173.82 ± 31.66 ^b^	308.48 ± 50.17 ^c^	476.42 ± 51.44 ^d^	ND	ND	ND	506.02 ± 91.70 ^d^	ND	ND	ND	ND	ND
113	(S)-(-)-Limonene	ND	0.68 ± 0.10 ^a^	0.83 ± 0.11 ^a^	1.20 ± 0.07 ^b^	ND	ND	ND	1.36 ± 0.19 ^b^	ND	ND	ND	ND	ND
114	Calamenene	0.16 ± 0.02	ND	ND	ND	ND	ND	ND	ND	ND	ND	ND	ND	ND
115	Glycerol	ND	ND	ND	ND	ND	ND	ND	15.82 ± 1.36 ^a^	ND	ND	ND	ND	48.51 ± 4.21 ^b^
Total		117.74 ± 15.11 ^a^	2899.00 ± 444.00 ^bc^	4549.01 ± 651.07 ^de^	7711.46 ± 794.92 ^g^	2359.22 ± 256.97 ^b^	13,843.26 ± 1078.26 ^i^	16,019.27 ± 1909.61 ^j^	3199.93 ± 496.19 ^bc^	814.87 ± 28.26 ^a^	5980.14 ± 536.39 ^f^	5395.81 ± 526.45 ^ef^	3643.04 ± 378.49 ^cd^	11,048.44 ± 1486.71 ^h^

Note: All values are means of triplicate determinations ± SD; “ND”, not detected; different letters indicate significant differences in compound content on the same row.

In both types of fermentation broth, the content of alcoholic compounds was highest in each stage, with 11,821.77 µg/L and 7352.84 µg/L in FMQFB and IQFB, respectively, on the 27th day of fermentation. This was inconsistent with previous studies reporting ester as having the highest content in the Huangjiu fermented from corn [18], which may be related to the differences in raw materials and fermentation processes. Among them, isoamyl alcohol and phenyl alcohol dominated, with their contents ranging from 759.20 to 5907.94 µg/L and 583.96 to 3788.04 µg/L in FMQFB, respectively, while ranging from 240.26 to 3890.74 µg/L and 223.08 to 2123.31 µg/L in IQFB, respectively, and they had the highest content on the 27th day of fermentation. There were significant differences in the content of VACs at different fermentation stages. Both of them, which can be formed by amino acids during the processes of biotransformation [38], are an important part of the aroma of Huangjiu [39]. Accordingly, the content of amino acids in FMQFB may be higher than that in IQFB. The partial alcohol compounds provide a certain contribution to floral fragrance of Huangjiu [39]. Esters are the second most abundant compounds in both fermentation broths, with concentrations of 3558.05 µg/L and 2752.34 µg/L in FMQFB and IQFB on the 27th day, respectively. The ester compound with the highest content was ethyl acetate, which gradually increased in the middle and late stages of FMQFB fermentation and reached a content of approximately 1898.62 µg/L at the end stage. Starting from the 5th day of IQFB fermentation, it was detected that the content was approximately 1938.47 µg/L at the end of fermentation. Compared to FMQFB, ethyl acetate was produced earlier and at higher contents in IQFB at the end, but the final content was not significantly different. In addition, compounds such as ethyl palmitate, ethyl decanoate, ethyl caprylate, and phenethyl acetate were found to be higher in FMQFB, meanwhile, isoamyl acetate, ethyl caprylate, ethyl caprylate, and phenethyl acetate were discovered to be higher in IQFB. Hence, one can see that the majority of ester aroma compounds in Huangjiu were ethyl esters, which were mainly produced by the esterification reaction of ethanol catalyzed by acetyltransferase [40,41]. Most of the esters produce fruity or floral aromas, which play a positive role in the aroma of Huangjiu. At the end of fermentation, the levels of acids in FMQFB and IQFB were 540.55 µg/L and 741.05 µg/L, respectively. The content of acetic acid, isobutyric acid, and nonanoic acid remained high during the fermentation process and gradually increased as the fermentation proceeded. On the 27th day, the content of acetic acid in FMQFB and IQFB reached 435.98 µg/L and 598.69 µg/L, respectively. Acetic acid has the odor of vinegar, which is produced in Huangjiu by alanine [42]. Most of the fatty acids in Huangjiu are formed by microbial fermentation [43], but they decreased significantly in the post-fermentation stage, which may had a bearing on the involvement of acids in esterification reactions. Acids play a role in harmonizing the aromas of Huangjiu, thereby reducing bitterness and unpleasant odors and enhancing the fullness of the wine.

In general, the content of various volatile aroma compounds showed an increasing trend in the early period and a fluctuating increase in the middle and late period, reaching the highest value on the 27th day of fermentation. The content of esters such as ethyl acetate, isoamyl acetate, ethyl caproate, ethyl heptanoate, and ethyl nonoate was significantly higher in IQFB compared to FMQFB. The categories of VACs in IQFB were higher than in FMQFB and the content was lower than in FMQFB on the 27th day. Moreover, compared with esters, alcohols, and acids at the same stage, other compounds such as aldehydes and ketones had lower content in the two fermentation broths, but they also contributed to the aroma of Huangjiu. It was the difference in the type and content of VACs in FMQFB and IQFB that led to differences in their aromas.

### 3.6. Sensory Evaluation of Fermentation Broths

**Figure 5:**
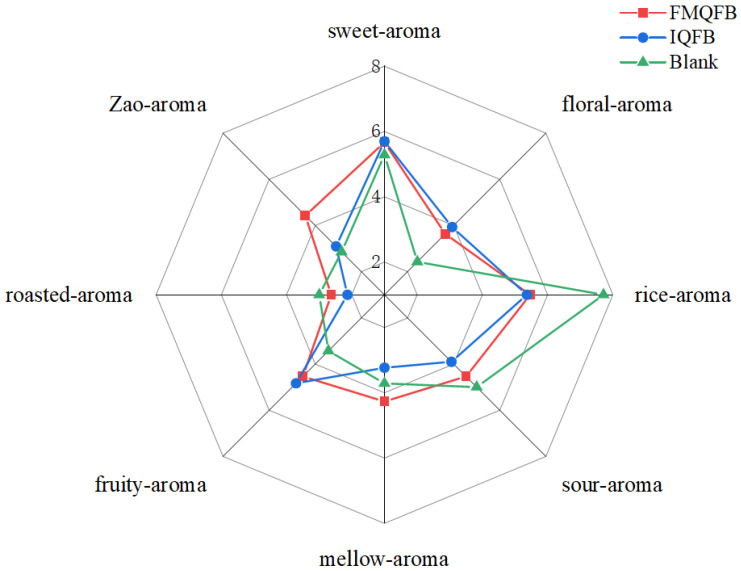
Outline of sensory evaluation of aroma of fermented Huangjiu.

In order to further understand the aroma differences between FMQFB and IQFB, sensory evaluations on the aroma characteristics of the blank sample and two types of Huangjiu were conducted by 15 sensory evaluation members, as shown in Figure 5.

From Figure 5, it can be seen that the blank sample had significantly lower floral and fruit aromas than FMQFB and IQFB, while the rice, roasted, and sour aromas were higher than in the two types of Huangjiu. The intensities of fruity and floral aroma of IQFB were obviously stronger, yet the intensities of Zao aroma (similar to fermented grain), roasted aroma, mellow aroma, and sour aroma were less than in FMQFB. Ethyl hexanoate and isoamyl acetate mainly contributed to the fruity aroma of Huangjiu fermentation broths [14]. Based on the quantitative analysis of VACs, the concentration of them in IQFB was significantly higher than that in FMQFB, and the more obvious fruity aroma was also demonstrated by sensory evaluation. The Zao aroma in FMQFB was outstandingly higher than that in IQFB, which has been found to probably be produced by the combined influence of several compounds [42]. The content of alcohol compounds in IQFB was lower than that in FMQFB, which was also the major causation for the decrease in the alcohol aroma of IQFB. Pyrazine was a prominent contributor to roasted aroma [42]. Nevertheless, it was not detected in either fermentation broth on the 27^th^ day and, at the same time, it also had the lowest descriptor score among the eight aroma attributes in the sensory evaluation. Based on our previous work, it was found that *Wickerhamomyces anomalus* could increase the content of isoamyl acetate [44], which was added to IQ, resulting in an improved fruit aroma in IQFB. This was consistent with the results of quantitative analysis of VACs. However, the intensities of Zao aroma, roasted aroma, mellow aroma, and sour aroma were reduced.

### 3.7. Association between Microorganisms and Flavor Compounds

**Figure 6:**
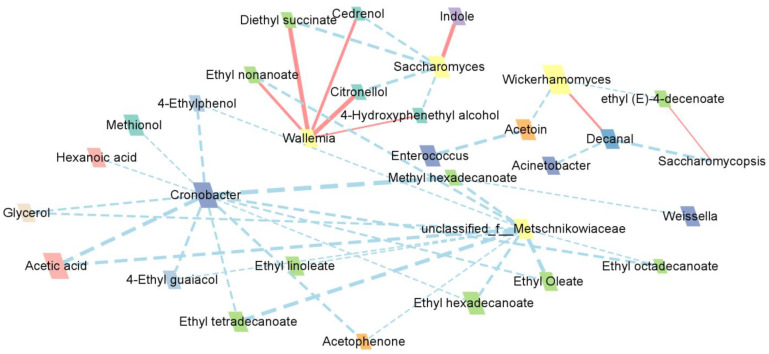
Relationships network between important microbial communities (abundance >0.1%) and aroma in IQFB.

To further explore the relationship between microbial communities and aroma, the correlation coefficients between microorganisms with > 0.1% abundance and VACs were calculated using Spearman correlation analysis. There were 352 positive correlations and 506 negative correlations, and a total of 858 correlations were obtained. Correlations with correlation coefficients |r| > 0.7 were visualized using Cytoscape, yielding 42 associations of microorganisms and VACs, shown in Figure 6, including 8 positive and 34 negative correlations. There were positive correlations between *Saccharomyces* and indole, *Saccharomycopsis* and ethyl (E)-4-decenoate, *Wickerhamomyces* and decanal, and *Wallemia* and ethyl nonanoate, diethyl succinate, citronellol, cedrenol, and 4-hydroxyphenethyl alcohol. *Wallemia* had been detected from a Maotai-flavor liquor brewing environment, being the absolute dominant fungal genus in each round and having a strong positive correlation with the growth metabolism of *Aspergillus* [45]. This produced a number of extracellular enzymes that led to the formation of aromatic compounds [46,47]. Among the fungi, *Saccharomycopsis*, which had the highest abundance, also showed a negative correlation with decanal. Meanwhile, *Wickerhamomyces*, which was much more abundant, was associated with ethyl (E)-4-decenoate and acetoin. The high predominance of *Cronobacter* was negatively correlated with most compounds as well as highly correlated with the metabolism of long-chain fatty acid esters such as ethyl tetradecanoate, methyl hexadecanoate, ethyl hexadecanoate, ethyl octadecanoate, and ethyl oleate. The unclassified_f__Metschnikowiaceae in fungi was also strongly associated with long-chain fatty acid esters.

### 3.8. Analysis of Functional Microbial Metabolic Pathways

**Figure 7:**
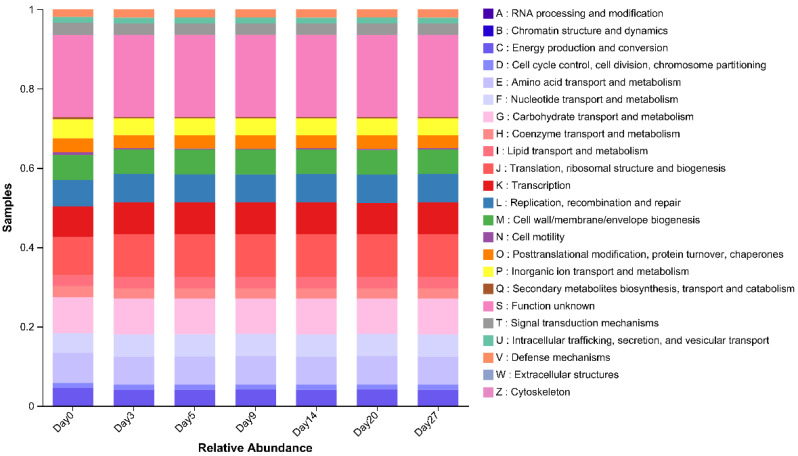
Bar graph of COG function classification.

A total of 23 categories were classified by COG function classification, shown in Figure 7. The highest abundance was noted for S (function unknown), with maximum and minimum values on day 27 (20.79%) and day 0 (20.71%), respectively. This was followed by J (translation, ribosomal structure, and biogenesis), which had the largest and smallest abundance on day 3 (10.84%) and day 0 (9.74%). Meanwhile, G (carbohydrate transport and metabolism), E (replication, recombination, and repair), and L (transcription) were also functional classifications with high abundance, accounting for 8.93–8.97%, 7.03–7.53%, and 6.68–7.14%, correspondingly. Metabolism, the most common of all functional types, had a high share of about 32%. Classification of functions linked to metabolism included G (carbohydrate transport and metabolism), E (amino acid transport and metabolism), F (nucleotide transport and metabolism), P (inorganic ion transport and metabolism), I (lipid transport and metabolism), H (coenzyme transport and metabolism), and Q (secondary metabolite biosynthesis, transport, and catabolism). The function of E, G, and F played an essential role in the metabolism of carbohydrates, amino acids, and nucleotides in microorganisms, and they were the three categories with the highest abundance of metabolic functions, representing about 82.38% overall.

**Figure 8:**
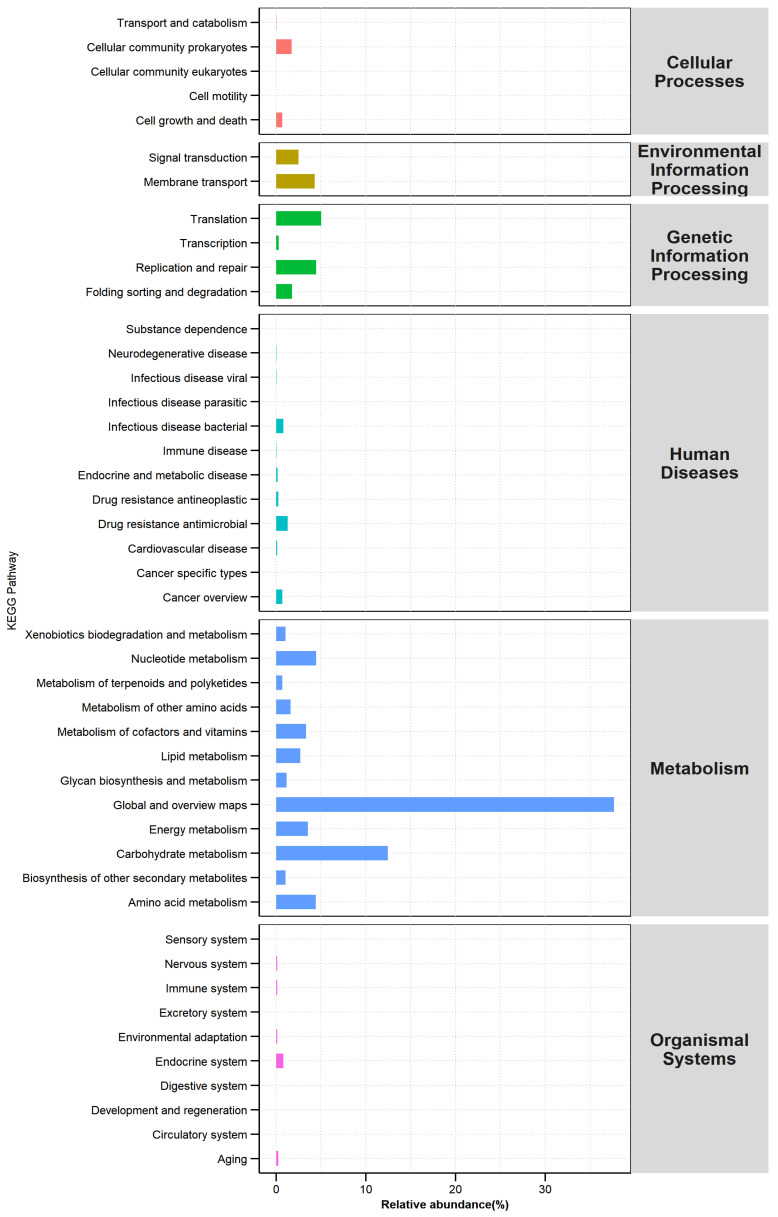
KEGG pathway classification abundance map.

Based on the KEGG database, 6, 45, and 340 level were classified to 1, 2, and 3 pathways, respectively (Figure 8). For pathways 1, a high percentage of metabolism was found at 74.18%, while genetic information processing was the second most abundantly annotated gene with 11.58%, followed by environmental information processing with 6.78%. Within the secondary pathways, the most abundantly annotated genes in IQFB were global and overview maps in metabolism and carbohydrate metabolism and translation in genetic information processing, accounting for 37.69%, 12.45%, and 5.02%. In all, there were 18 secondary metabolic pathways contributing more than 1%. In addition to the above, nucleotide metabolism (4.48%), replication and repair (4.47%), and amino acid metabolism (4.41%) were the prominent metabolic functions of microorganisms in IQFB. In 3 pathways, in which the 156 annotated genes were contained in metabolic pathways, ko01230 (biosynthesis of amino acids), ko00520 (amino sugar and nucleotide sugar metabolism), and ko00010 (glycolysis/gluconeogenesis) were also essential microbial metabolic pathways in fermentation broths. This was completely distinct from the major metabolic pathways of nonvolatile chemical constituents in Huangjiu, including biosynthesis of cofactors, biosynthesis of amino acids, and ABC transport [48]. From this, we can have a clear and intuitive understanding of the function of microorganisms in the fermentation process.

## 4. Conclusions

In this paper, by using FMQ as seed Qu, *Saccharomyces cerevisiae* solution and *Wickerhamomyces anomalus* solution were added to produce IQ, and brewing trials were conducted using these two types of Qu. Microbial diversity analysis of IQFB at different brewing stages revealed that *Pediococcus*, *Cronobacter*, *Enterococcus*, *Weissella*, and *Acinetobacter* and *Saccharomycopsis*, *Wickerhamomyces*, and *Saccharomyces* were the dominant bacterial and fungal groups, respectively. Compared to fungi, bacteria had a greater diversity and abundance. 92 and 74 compounds were identified by HS-SPME combined with GC-MS for the fermentation broths of the two Qu, correspondingly. At the end of fermentation, more species of VACs were found in IQFB than FMQFB and the content was lower than in FMQFB. Among them, the content of esters such as ethyl acetate, isoamyl acetate, ethyl caproate, ethyl heptanoate, and ethyl nonanoate in IQFB was significantly higher compared with FMQFB. According to the sensory evaluation, it was shown that the addition of pure yeast to Qu could enhance the fruit and floral aromas of Huangjiu. A network of relationships between important microbial communities (abundance > 0.1%) and aroma in IQFB was established, and 858 correlations could be gained. The functions of microbial communities were predicted, finding global and overview maps and carbohydrate metabolism to be the most dominant. This will provide reference data for stabilizing the quality of Qu and improving the quality of Huangjiu, which could facilitate further regulation of the fermentation process of Huangjiu by microbiological measures.

## Figures and Tables

**Table 1:** Content of physicochemical indexes in fermented of Huangjiu.

Time	Total Acid (g/L)		Total Sugar (g/L)		pH		Alcohol Content (%vol)	
	FMQFB	IQFB	FMQFB	IQFB	FMQFB	IQFB	FMQFB	IQFB
3 d	3.01 ± 0.03	2.18 ± 0.05 ***	266.00 ± 4.83	282.58 ± 5.25 *	3.70 ± 0.01	3.94 ± 0.01 ***	3.76 ± 0.06	3.46 ± 0.36
5 d	3.07 ± 0.08	2.72 ± 0.09 **	290.46 ± 11.28	295.15 ± 4.47	3.84 ± 0.01	3.92 ± 0.01 ***	5.89 ± 0.10	9.28 ± 0.35 ***
9 d	3.25 ± 0.07	2.46 ± 0.06 ***	152.85 ± 5.91	114.79 ± 1.03 ***	3.62 ± 0.01	3.83 ± 0.01 ***	7.41 ± 0.15	8.93 ± 0.19 ***
14 d	3.31 ± 0.11	2.70 ± 0.07 **	112.03 ± 0.62	83.81 ± 0.52 ***	3.76 ± 0.01	3.92 ± 0.05 **	11.62 ± 0.12	10.22 ± 0.71 *
20 d	3.47 ± 0.03	2.43 ± 0.03 ***	81.55 ± 1.05	56.93 ± 0.36 ***	3.76 ± 0.01	3.92 ± 0.01 ***	13.92 ± 0.36	12.28 ± 0.18 **
27 d	3.57 ± 0.02	2.86 ± 0.05 ***	67.51 ± 1.11	40.60 ± 0.65 ***	3.82 ± 0.01	3.98 ± 0.01 ***	14.79 ± 0.13	14.73 ± 0.44

NOTE: * indicates the significance of the same index for different fermentation broths on the same day; *, significant (*p* < 0.05); **, highly significant (*p* < 0.01); ***, very highly significant (*p* < 0.001).

## Data Availability

The authors declare that all data supporting the findings of this study are available within the paper. The datasets generated and analyzed during the current study are also available from the corresponding author upon request.

## References

[1] Yang Y.J., Hu W.Y., Xia Y.J., Mu Z.Y., Tao L.R., Song X., Zhang H., Ni B., Ai L.Z. (2020). Flavor Formation in Chinese Rice Wine (Huangjiu): Impacts of the Flavor-Active Microorganisms, Raw Materials, and Fermentation Technology. Front. Microbiol..

[2] Zhu Y.B., Zhang J.H., Shi Z.P., Mao Z.G. (2004). Optimization of operating conditions in rice heat blast process for Chinese rice wine production by combinational utilization of neural network and genetic algorithms. J. Inst. Brew..

[3] Luo T., Fan W., Yan X. (2012). Characterization of Volatile and Semi-Volatile Compounds in Chinese Rice Wines by Headspace Solid Phase Microextraction Followed by Gas Chromatography Mass Spectrometry. J. Inst. Brew..

[4] Wang J.L., Zhang Y., YU Q. (2011). Research progress of flavoring components in Yellow Rice Wine. Liquor Mak. Sci. Technol..

[5] Jiao A.Q., Xu X.M., Jin Z.Y. (2017). Research progress on the brewing techniques of new-type rice wine. Food Chem..

[6] Xie G.F., Zheng H.J., Qiu Z.L., Lin Z.C., Peng Q., Bealu G.D., Elsheery N.I., Lu Y., Shen C., Fu J.W. (2021). Study on relationship between bacterial diversity and quality of Huangjiu (Chinese Rice Wine) fermentation. Food Sci. Nutr..

[7] Zhang K.Z., Li Q., Wu W.C., Yang J.G., Zou W. (2019). Wheat Qu and Its Production Technology, Microbiota, Flavor, and Metabolites. J. Food Sci..

[8] Hou Z.H., Ning H.R., Nie S., Zhang X., Zhang Y., Cui S.H. (2013). Research progress in wine starter in the world. Spec. Wild Econ. Anim. Plant Res..

[9] Xie G.F., Li W.J., Lu J., Cao Y., Fang H., Zou H.J., Hu Z.M. (2007). Isolation and identification of representative fungi from Shaoxing rice wine wheat Qu using a polyphasic approach of culture-based and molecular-based methods. J. Inst. Brew..

[10] Ran Y.Z., Zhang Y., Cai G.H., Mao Y.G., Yuan J.C., Yu J.S. (2009). A new method to study the effects of wheat starter on the flavor of yellow rice wine. Liquor-Mak. Sci. Technol..

[11] Guo W., Zhou J.B., Fang S.L., Yu C.W., Chen M.B. (2015). Application & Research progress in intensified Daqu. Liquor-Mak. Sci. Technol..

[12] Wei H.L., Zhou J.H., Liu F.C., Guan J., Deng Q.J., Chen M.B., Fang S.L. (2017). Screening of Rhizopus from glutinous rice wine starter and research on fortified Qu. China Brew..

[13] Cui D.Q., Luo X.Y., Ban S.D., Huang W., Zhang Y.H., Zhao H.J., Wang X.D. (2022). Preparation of fortified Daqu and its application in Moutai-flavor Baijiu. Food Ferment. Ind..

[14] Chen S., Xu Y., Qian M.C. (2018). Comparison of the aromatic profile of traditional and modern types of Huang Jiu (Chinese rice wine) by aroma extract dilution analysis and chemical analysis. Flavour Fragr. J..

[15] Ye Y.T., Wang L.X., Zhan P., Tian H.L., Liu J.S. (2022). Characterization of the aroma compounds of Millet Huangjiu at different fermentation stages. Food Chem..

[16] Tempere S., Marchal A., Barbe J.C., Bely M., Masneuf-Pomarede I., Marullo P., Albertin W. (2018). The complexity of wine: Clarifying the role of microorganisms. Appl. Microbiol. Biotechnol..

[17] Yan Y., Sun L.P., Xing X., Wu H.J., Lu X., Zhang W., Xu J.L., Ren Q. (2022). Microbial succession and exploration of higher alcohols-producing core bacteria in northern Huangjiu fermentation. AMB Express.

[18] Ren Q., Sun L.P., Sun Z.B., Liu Q.S., Lu X., Li Z.P., Xu J.L. (2020). Bacterial succession and the dynamics of flavor compounds in the Huangjiu fermented from corn. Arch. Microbiol..

[19] Ren Q., Sun L.P., Wu H.J., Wang Y.S., Wang Z.W., Zheng F.P., Lu X., Xu J.L. (2019). The changes of microbial community and flavor compound in the fermentation process of Chinese rice wine using Fagopyrum tataricum grain as feedstock. Sci. Rep..

[20] Huang Z.R., Guo W.L., Zhou W.B., Li L., Xu J.X., Hong J.L., Liu H.P., Zeng F., Bai W.D., Liu B. (2019). Microbial communities and volatile metabolites in different traditional fermentation starters used for Hong Qu glutinous rice wine. Food Res. Int..

[21] Wang J., Yu Y.G., Gao X.L., Jiang X.Y., Huang M.Q., Ye H., Wu J.H., Zhang J.L., Sun X.T., Wu Q. (2022). Succession patterns of aroma components during brewing process of broomcorn millet (*Panicum miliaceum* L.) Huangjiu. Food Res. Int..

[22] Yang Y.J., Ai L.Z., Mu Z.Y., Liu H.D., Yan X., Ni L., Zhang H., Xia Y.J. (2022). Flavor compounds with high odor activity values (OAV > 1) dominate the aroma of aged Chinese rice wine (Huangjiu) by molecular association. Food Chem..

[23] Liu H.J., Wang J., Wang Z.C., Huang M.Q., Liu Y.G., Wu J.H., Zhang J.L. (2022). Characterization of volatile compounds in Fangxian Huangjiu by two-dimensional gas chromatography-mass spectrometry. Food Ferment. Ind..

[24] Yan Y., Chen H.Y., Sun L.P., Zhang W., Lu X., Li Z.P., Xu J.L., Ren Q. (2022). The changes of microbial diversity and flavor compounds during the fermentation of millet Huangjiu, a traditional Chinese beverage. PLoS ONE.

[25] Zhang W.D., Ren Q., Wang Z.C., Liu H.J., Huang M.Q., Wu J.H., Sun B.G. (2022). Analysis of the Microbial Community Structure and Volatile Metabolites of JIUYAO in Fangxian, China. Fermentation.

[26] Yu H.Y., Li Q.W., Guo W., Chen C., Ai L.Z., Tian H.X. (2023). Dynamic analysis of volatile metabolites and microbial community and their correlations during the fermentation process of traditional Huangjiu (Chinese rice wine) produced around Winter Solstice. Food Chem. X.

[27] Lee E., Nam K.T., Lee K.W., Lee S.R. (2020). Pediococcus spp.-fermented chicken meat for dogs. J. Anim. Sci. Technol..

[28] Rathee K., Dhull V., Dhull R., Singh S. (2016). Biosensors based on electrochemical lactate detection: A comprehensive review. Biochem. Biophys. Rep..

[29] Xiang F.S., Liu X.T., Dai C.Y., Zhang Z.D., Zhuang G. (2020). Analysis of microbial diversity of rice wine in Xuanen area based on MiSeq high-throughput sequencing technology. Sci. Technol. Food Ind..

[30] Yang Y.J., Xia Y.J., Hu W.Y., Tao L.R., Liu H.D., Xie C.L., Bai W.D., Ai L.Z. (2021). Soaking induced discrepancies in oenological properties, flavor profiles, microbial community and sensory characteristic of Huangjiu (Chinese rice wine). LWT Food Sci. Technol..

[31] Romano P., Fiore C., Paraggio M., Caruso M., Capece A. (2003). Function of yeast species and strains in wine flavour. Int. J. Food Microbiol..

[32] Chen C., Liu Y., Tian H.X., Ai L.Z., Yu H.Y. (2020). Metagenomic analysis reveals the impact of JIUYAO microbial diversity on fermentation and the volatile profile of Shaoxing-jiu. Food Microbiol..

[33] Yuan H.S., Liu G.L., Bai W.D., Liang J.L. (2023). Research progress on the application of ester-producing yeast in fermented food. China Brew..

[34] Fan G.S., Teng C., Xu D., Fu Z.L., Minhazul Karim A.H.M., Wu Q.H., Liu P.X., Yang R., Li X.T. (2019). Enhanced production of ethyl acetate using co-culture of Wickerhamomyces anomalus and Saccharomyces cerevisiae. J. Biosci. Bioeng..

[35] Kim H.R., Kim J.H., Bai D.H., Ahn B. (2012). Feasibility of Brewing Makgeolli Using Pichia anomala Y197-13, a Non-Saccharomyces cerevisiae. J. Microbiol. Biotechnol..

[36] Liao P.F., Wang S., Wang Z., Chen L., Lu W., Sun J.Y., Li H.H., Zhao D.R., Wang B.W., Sun B.G. (2023). Analysis of Aroma Components of Five Steamed Grains for Production of Nongxiangxing Baijiu by Simultaneous Distillation and Extraction combined with Gas Chromatography-Mass Spectrometry. Food Sci..

[37] Zhang H.X., Wang L., Wang H.Y., Yang F., Chen L.Q., Hao F., Lv X.B., Du H., Xu Y. (2021). Effects of initial temperature on microbial community succession rate and volatile flavors during Baijiu fermentation process. Food Res. Int..

[38] Hazelwood L.A., Daran J.M., van Maris A.J.A., Pronk J.T., Dickinson J.R. (2008). The ehrlich pathway for fusel alcohol production: A century of research on Saccharomyces cerevisiae metabolism. Appl. Environ. Microbiol..

[39] Zhou Z.L., Ji Z.W., Liu S.P., Han X., Zheng F.P., Mao J. (2019). Characterization of the volatile compounds of huangjiu using comprehensive two-dimensional gas chromatography coupled to time of flight mass spectrometry (GC × GC-TOFMS). J. Food Process Preserv..

[40] Erten H., Tanguler H., Cakiroz H. (2007). The effect of pitching rate on fermentation and flavour compounds in high gravity brewing. J. Inst. Brew..

[41] Xu Y.Q., Wu M.Q., Zhao D., Zheng J., Dai M.Q., Li X.T., Li W.W., Zhang C.N., Sun B.G. (2023). Simulated Fermentation of Strong-Flavor Baijiu through Functional Microbial Combination to Realize the Stable Synthesis of Important Flavor Chemicals. Foods.

[42] Wang J., Yuan C.J., Gao X.L., Kang Y.L., Huang M.Q., Wu J.H., Liu Y.P., Zhang J.L., Li H.H., Zhang Y.Y. (2020). Characterization of key aroma compounds in Huangjiu from northern China by sensory-directed flavor analysis. Food Res. Int..

[43] Li J.S. (2001). Sources of color components, aroma components and taste components in yellow rice wine. Liquor-Mak. Sci. Technol..

[44] Zhang W.D. (2022). Analysis of Microbial Diversity and Characteristics of Fangxian JIUYAO and Preparation of Intensified JIUYAO. Master’s Thesis.

[45] Li Y.Y., Hu X.X., Huang Y.G. (2021). Analysis of the diversity of fungal flora in Maotai-flavor liquor brewing environment in Maotai Town. Food Sci. China.

[46] Sonia K.G., Chadha B.S., Saini H.S. (2005). Sorghum straw for xylanase hyper-production by Thermomyces lanuginosus (D_2_W_3_) under solid-state fermentation. Bioresour. Technol..

[47] Lv X.C., Huang Z.Q., Zhang W., Rao P.F., Ni L. (2012). Identification and characterization of filamentous fungi isolated from fermentation starters for Hong Qu glutinous rice wine brewing. J. Gen. Appl. Microbiol..

[48] Wang J., Wang D.Q., Huang M.Q., Sun B.G., Ren F.Z., Wu J.H., Meng N., Zhang J.L. (2023). Identiffcation of nonvolatile chemical constituents in Chinese Huangjiu using widely targeted metabolomics. Food Res Int..

